# Human naive epiblast cells possess unrestricted lineage potential

**DOI:** 10.1016/j.stem.2021.02.025

**Published:** 2021-06-03

**Authors:** Ge Guo, Giuliano Giuseppe Stirparo, Stanley E. Strawbridge, Daniel Spindlow, Jian Yang, James Clarke, Anish Dattani, Ayaka Yanagida, Meng Amy Li, Sam Myers, Buse Nurten Özel, Jennifer Nichols, Austin Smith

**Affiliations:** 1 Wellcome-MRC Cambridge Stem Cell Institute, Jeffrey Cheah Biomedical Centre, University of Cambridge, Cambridge CB2 0AW, UK; 2 Department of Physiology, Development and Neuroscience, University of Cambridge, Cambridge CB2 1GA, UK; 3 Department of Biochemistry, University of Cambridge, Cambridge CB2 1QR, UK; 4 Living Systems Institute, University of Exeter, Exeter EX4 4QD, UK; 5 Guangzhou Institutes of Biomedicine and Health (GIBH), Chinese Academy of Sciences, Guangzhou 510530, China

**Keywords:** pluripotency, epiblast, embryonic stem cells, trophoblast, embryo, blastocyst, lineage segregation, differentiation

## Abstract

Classic embryological experiments have established that the early mouse embryo develops via sequential lineage bifurcations. The first segregated lineage is the trophectoderm, essential for blastocyst formation. Mouse naive epiblast and derivative embryonic stem cells are restricted accordingly from producing trophectoderm. Here we show, in contrast, that human naive embryonic stem cells readily make blastocyst trophectoderm and descendant trophoblast cell types. Trophectoderm was induced rapidly and efficiently by inhibition of ERK/mitogen-activated protein kinase (MAPK) and Nodal signaling. Transcriptome comparison with the human embryo substantiated direct formation of trophectoderm with subsequent differentiation into syncytiotrophoblast, cytotrophoblast, and downstream trophoblast stem cells. During pluripotency progression lineage potential switches from trophectoderm to amnion. Live-cell tracking revealed that epiblast cells in the human blastocyst are also able to produce trophectoderm. Thus, the paradigm of developmental specification coupled to lineage restriction does not apply to humans. Instead, epiblast plasticity and the potential for blastocyst regeneration are retained until implantation.

## Highlights

• Human naive pluripotent stem cells form blastocyst trophectoderm directly
• Unlike in mouse, human naive epiblast regenerates trophectoderm
• Inhibition of ERK and Nodal drives trophectoderm differentiation
• Potency changes from trophectoderm to amnion during pluripotency progression

## Graphical abstract

**Figure:**
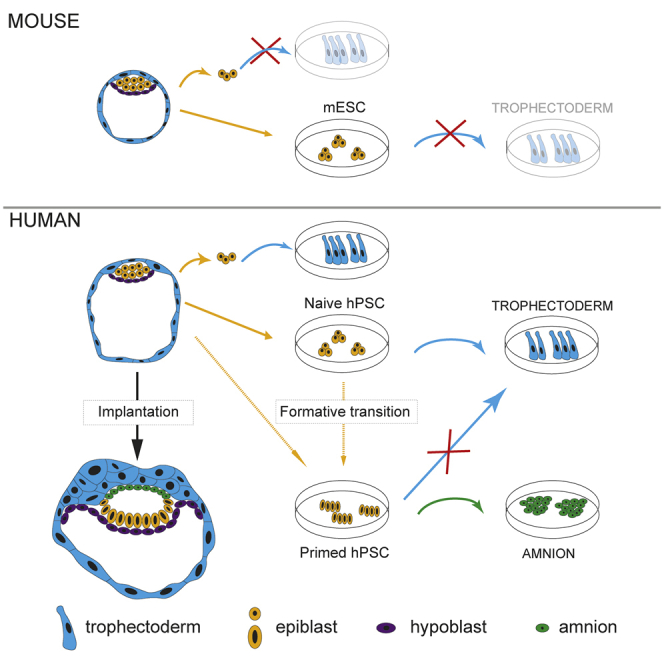

## Teaser

Human pluripotent stem cells (hPSCs) exist in naive or primed states, but it has been unclear whether these correspond to distinct developmental potencies. Here, Guo et al. show that naive hPSCs differentiate into trophectoderm, the founder tissue of the placenta, whereas primed hPSCs have lost this potential and instead form the amnion.

## Introduction

Delamination of epithelial trophectoderm is the first differentiation event in mammalian embryos. Trophectoderm is a cell lineage evolved to mediate blastocyst formation and uterine implantation and, later, to produce components of the placenta. Trophectoderm derivatives also provide morphogenetic signals that pattern the early embryo. Following fertilization and early cleavage divisions, blastomeres divide asymmetrically to form trophectoderm and inner cell mass (ICM). Classic studies in mouse embryos have established that topological segregation of trophectoderm and ICM is followed rapidly by fate restriction so that by the mid-blastocyst (late 32-cell) stage, ICM cells can no longer make trophectoderm (Gardner, 1983; Nichols and Gardner, 1984). Lineage restriction is reflected in consolidation of distinct molecular identities (Posfai et al., 2017). Subsequently, a second binary fate decision resolves the ICM into epiblast and hypoblast (primitive endoderm) (Chazaud et al., 2006; Gardner and Rossant, 1979; Plusa et al., 2008; Saiz et al., 2016). These observations have given rise to a textbook model of sequential lineage bifurcations at the onset of mammalian embryo development (Rossant, 2018).

Mouse embryonic stem cells (ESCs) are cell lines derived directly from the naive pre-implantation epiblast (Brook and Gardner, 1997; Evans and Kaufman, 1981; Martin, 1981; Nichols et al., 2009). Over prolonged expansion *in vitro*, they retain global transcriptome proximity to their tissue stage of origin (Boroviak et al., 2014). Functionally, they can contribute massively to all embryo tissues in chimeras but do not make appreciable contributions to trophectoderm derivatives (Beddington and Robertson, 1989; Bradley et al., 1984; Nagy et al., 1993; Posfai et al., 2021), in line with the paradigm of early segregation.

Trophectoderm versus ICM determination in the developing mouse blastocyst is underpinned by mutually exclusive and antagonistic expression of the transcription factors Oct4 and Cdx2 (Strumpf et al., 2005). ESCs can be made to transdifferentiate into trophectoderm-like cells by forced expression of *Cdx2* or deletion of *Oct4* (Niwa et al., 2005). Expression of other trophectoderm lineage transcription factors, such as Tfap2c (Adachi et al., 2013), or demethylation and upregulation of Elf5 (Ng et al., 2008) also provokes transdifferentiation into trophectoderm. Detailed characterization, however, has revealed that, although cells with morphological features and some markers of trophoblast are obtained, functional phenotypes are not established properly (Cambuli et al., 2014). Moreover, depletion of Nanog, a central component of the ESC transcription factor network, destabilizes naive identity but results in differentiation to hypoblast (Chambers et al., 2007; Mitsui et al., 2003), indicating that trophectoderm is not a “default” program. Thus, trophectoderm lineage restriction appears to be hard-wired in mouse epiblast and ESCs.

Human pluripotent stem cells (hPSCs) (Takahashi et al., 2007; Thomson et al., 1998; Yu et al., 2007) differ from mouse ESCs and are considered to represent post-implantation epiblast (Rossant, 2015). hPSCs have been reported to differentiate into trophoblast-like cells upon treatment with bone morphogenetic protein (BMP) (Amita et al., 2013; Xu et al., 2002). Generation of a pre-implantation lineage by stem cells that have a post-implantation identity (Nakamura et al., 2016; O’Leary et al., 2012) is surprising and without developmental precedent. Furthermore, BMP is not involved in trophectoderm specification in the human blastocyst (De Paepe et al., 2019), and the induced cells *in vitro* do not fulfill stringent criteria for trophoblast identity (Bernardo et al., 2011; Lee et al., 2016). More recently, extended potential hPSCs (hEPSCs) have been described and reported to form trophoblast-like cells, also in a BMP-dependent manner (Gao et al., 2019; Yang et al., 2017). However, the developmental authenticity of EPSCs or their trophoblast-like progeny has yet to be ascertained (Posfai et al., 2021).

Culture conditions have now been developed (Guo et al., 2017; Takashima et al., 2014; Theunissen et al., 2014) that support self-renewal of hPSCs with transcriptomic and other features of naive pluripotency (Bredenkamp et al., 2019b; Dong et al., 2019; Nakamura et al., 2016; Stirparo et al., 2018). Availability of stem cell counterparts of naive epiblast provides an opportunity for experimental interrogation of lineage restriction in early human development. Here we investigate naive cell propensity to produce trophectoderm and find that this reflects the plasticity of human pre-implantation epiblast unlike conventional post-implantation-stage hPSCs.

## Results

### Human naive stem cells can enter the trophectoderm lineage

**Figure 1:**
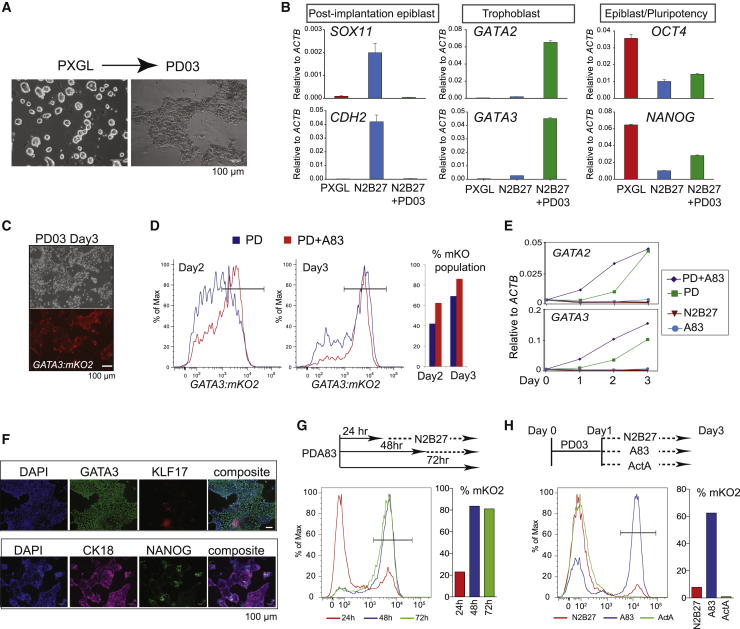
Trophectoderm formation (A) Images of naive stem cells and cells differentiating in PD after 3 days. (B) qRT-PCR assay for post-implantation epiblast, trophectoderm, and core pluripotency markers after 5 days under the indicated conditions. Error bars are from technical duplicates. (C) Phase and fluorescence images of GATA3:mKO2 reporter cells after 3 days in PD only. (D) Flow cytometry analysis of GATA3:mKO2 cells exposed to PD+A83 for the indicated periods. (E) qRT-PCR assay of *GATA2* and *GATA3* expression over time under the indicated conditions. Error bars are from technical duplicates. (F) Immunostaining for the trophectoderm markers GATA3 and cytokeratin 18 (CK18) and the naive markers KLF17 and NANOG after 3 days in PD+A83. (G) Flow cytometry analysis of GATA3:mKO2 cells treated with PD+A83 for the indicated periods, followed by N2B27. (H) Flow cytometry analysis of GATA3:mKO2 cells treated with PD for 24 h, followed by transfer to A83 or Activin A for 48 h.

Mouse ESCs self-renew efficiently in the presence of LIF (leukemia inhibitory factor) and the MEK (mitogen activated protein kinase kinase) inhibitor PD0325901 (PD) (Dunn et al., 2014; Ying et al., 2008). Stable propagation of human naive stem cells additionally requires the atypical protein kinase C inhibitor Gö6983 and blockade of the Wnt pathway, a culture condition called PXGL (Bredenkamp et al., 2019b). While investigating the effects of individual inhibitors, we observed that culture in PD only resulted in differentiation into flattened epithelial cells (Figure 1A). To determine the character of these differentiated cells, we inspected early lineage markers. We did not detect upregulation of post-implantation epiblast markers that would signify formative transition (Rostovskaya et al., 2019; Figure 1B), nor were the hypoblast factors *GATA4*, *PDGFRA*, and *SOX17* expressed (Figure S1A). Instead, we observed marked upregulation of *GATA2* and *GATA3*, transcription factors characteristic of trophectoderm. Interestingly, the other inhibitors in PXGL, the XAV939 tankyrase inhibitor and Gö6983, individually reduced and together completely blocked expression of *GATA2* and *GATA3* (Figure S1B). We investigated the effect of culture in PD alone on three independent cell lines, including embryo-derived and chemically reset naive cells. Together with upregulation of GATA2 and GATA3, we saw induction of the trophectoderm markers TEAD3 and DAB2 (Figure S1C).

Apparent trophectoderm formation from human naive stem cells is surprising because mouse ESCs do not generate this lineage without genetic or epigenetic manipulation (Posfai et al., 2021). We cultured mouse ESCs in PD only and did detect weak induction of *GATA3* (Figure S1D). However, neither *GATA2* nor other trophectoderm genes were upregulated, consistent with inability to enter the lineage. Therefore, the plasticity of human naive cells in response to PD is species specific.

To monitor trophectoderm induction, we created a *GATA3:mKO2* knockin reporter line by CRISPR-Cas9-mediated homologous recombination in HNES1 naive cells (Figure 1C). Fluorescence was barely detected during self-renewal in PXGL or upon transfer to N2B27 but readily apparent in PD only. Naive stem cells displayed prominent SMAD2 phosphorylation (Figure S1E), indicative of autocrine Nodal stimulation (Rostovskaya et al., 2019). We added the inhibitor A83-01 (A83) to block Nodal signaling in PXGL (Figure S1F). After three passages, we detected reporter activation in a significant fraction of cells, coincident with increasing morphological differentiation (Figures S1G and S1H). We also saw cumulative increases in *GATA2* and *GATA3* mRNAs (Figure S1I). We combined PD and A83 (PD+A83) and saw that mKO2 bright cells appeared earlier and in greater numbers than in PD alone, reaching around 80% by day 3 (Figure 1D). We tested A83 alone but observed only rare activation of *GATA3:mKO2* (Figure S1J). Accordingly, mRNAs for *GATA3* and *GATA2* were upregulated more rapidly (Figure 1E). Live-cell imaging (Figure S1K; Videos S1 and S2) showed conversion of HNES1 cells over 60 h in PD+A83 into an mKO2-positive flat epithelial monolayer of trophectoderm-like cells. Immunostaining after 3 days showed that the majority of cells expressed GATA3 and the epithelial marker cytokeratin 18, exclusive from nests of cells positive for the naive factors NANOG and KLF17 (Figure 1F).

Video S1. Phase contrast time lapse of GATA3:mKO2 cells cultured in PD+A83 for 60 h, related to Figure 1

Video S2. Fluorescence time lapse of GATA3:mKO2 cells in Video S1, related to Figure 1

We investigated how long inhibitor treatment is required. We found that 48 h in PD+A83 was sufficient for robust induction of GATA3:mKO2 and trophectoderm gene expression (Figure 1G). Because A83 alone has little effect, we induced cells with PD for 24 h and then transferred them to A83 only. This treatment yielded over 60% mKO2-positive cells (Figure 1H). Conversely, exposure to activin almost entirely suppressed the emergence of positive cells.

These findings indicate that MEK/ERK inhibition is necessary and sufficient to potentiate trophectoderm specification and that NODAL inhibition promotes lineage entry.

### Trophectoderm differentiation and derivation of cytotrophoblast stem cells

**Figure 2:**
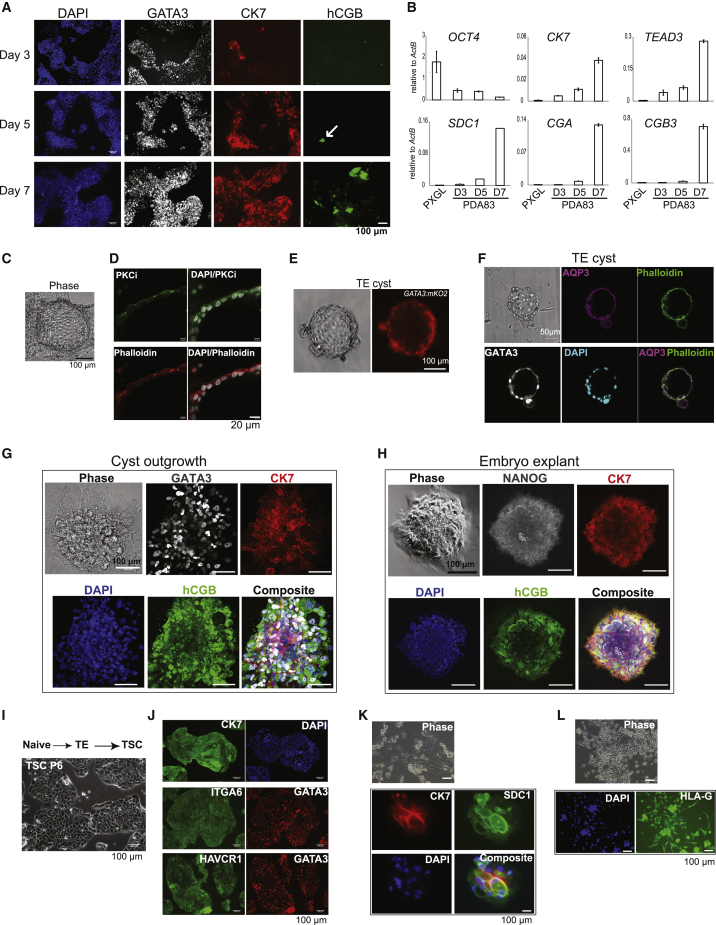
Trophoblast differentiation and TSC generation (A) Immunostaining for GATA3, CK7, and hCGB after the indicated days in PD+A83. (B) qRT-PCR assay of trophoblast marker expression at the indicated times. Error bars from technical duplicates. (C) Phase image of adherent epithelial cyst formed after PD+A83 treatment for 5 days. (D) Confocal image of adherent cyst immunostained for aPKCι. (E) GATA3-mKO-positive cyst formed in suspension culture in PD+A83 for 3 days. (F) Phalloidin and immunofluorescence staining of a cyst formed in suspension. (G) Immunostained outgrowth from a suspension cyst plated on laminin111-E8 for 5 days in N2B27. (H) Immunostained human blastocyst (E6) outgrowth after 5 days in N2B27 on laminin111-E8. (I) Phase contrast images of naive stem-cell-derived TSCs. (J) Immunostaining of naive stem-cell-derived TSCs at passage 5. (K and L) Phase contrast and immunostained images of naive stem-cell-derived TSCs differentiated under conditions for syncytiotrophoblast (K) or extravillous trophoblast (L).

During peri-implantation development, human trophectoderm gives rise to primary cytotrophoblast cells and syncytiotrophoblast. Cytokeratin 7 (CK7) serves as a pan-trophoblast marker, first expressed weakly in the late blastocyst and subsequently pronounced in cytotrophoblast cells (Deglincerti et al., 2016). During naive cell differentiation in PD+A83, CK7 was apparent in a few GATA3-positive cells on day 3 and then expressed widely and strongly from day 5 (Figure 2A). The syncytiotrophoblast marker β chorionic gonadotrophin (hCGB) was detected in rare clusters of positive cells on day 5 and became more prominent on day 7. qRT-PCR confirmed progressively increasing expression of CK7 together with TEAD3 and, on day 7, the presence of transcripts for the syncytiotrophoblast marker syndecan-1 (SDC1) and chorionic gonadotrophins (Figure 2B)

Trophectoderm is a transport epithelium that mediates formation of the blastocoel cavity by fluid uptake. The emergence in adherent culture of multiple cystic structures indicated functionality of the naive-cell-derived trophectoderm (Figure 2C). Consistent with a polarized epithelium, we detected expression of atypical protein kinase C iota (aPCKι) and PAR6B on the apical surface (Figures 2D and S2A). We investigated differentiation in PD+A83 in suspension culture and observed formation of cysts composed of mKO2-positive cells in at least 50% of wells (Figure 2E). Cyst epithelium displayed AQP3 (Figure 2F), the only aquaporin channel represented at appreciable levels in published human early embryo transcriptome data (Xiang et al., 2019). When transferred to dishes coated with laminin111-E8 in N2B27, cysts attached and formed outgrowths of GATA3- and CK7-positive cells, many of which also expressed hCGB (Figure 2G). The pattern of outgrowth and immunostaining mirrored that in explants of whole blastocysts (Figure 2H). We also detected expression of the extravillous trophoblast marker HLA-G in cyst outgrowths (Figure S2B).

Cytotrophoblast cells from placenta or blastocyst outgrowths can be converted *in vitro* into human trophoblast stem cells (TSCs) (Okae et al., 2018). We tested whether trophectoderm generated from naive cells in PD+A83 can give rise to expandable TSCs by replating into TSC medium. Numerous patches of cells with TSC-like morphology emerged within the first passage. After further passage without purification or colony picking, stable and morphologically relatively homogeneous epithelial cultures were established, as described for derivations of TSCs (Okae et al., 2018; Figure 2I). TSCs were derived from different naive cell lines in two independent experiments and showed similar marker expression as placental TSCs (Okae et al., 2018; Figure S2C). Immunostaining confirmed expression of CK7, ITGA6, HAVCR1, and GATA3 (Figure 2I). Naive-cell-derived TSCs could be induced to differentiate into hCGB- and SDC1-positive syncytiotrophoblast cells and HLA-G-expressing extravillous trophoblast, as described previously (Okae et al., 2018; Figures 2J–2L; Figure S2D).

Overall, these observations show that naive-cell-derived trophectoderm undergoes progressive differentiation into trophoblast lineage cells with a sequence and pattern that resemble peri-implantation development and that they can be converted readily into TSCs.

### Whole-transcriptome analysis of trophectoderm lineage differentiation

**Figure 3:**
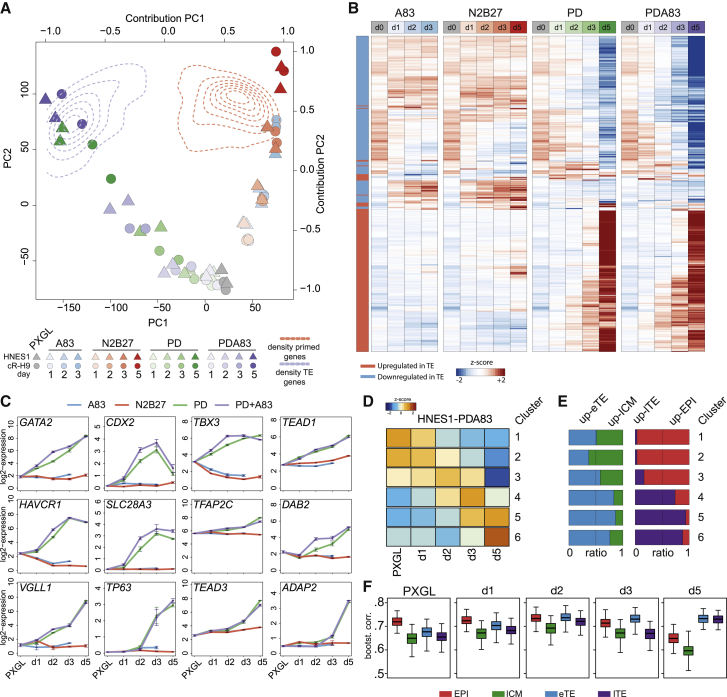
Whole-transcriptome analysis (A) PCA computed with all expressed protein-coding genes (log2 expression > 0, n = 19,450) and two-dimensional kernel density estimation of the contribution of genes with enriched expression in late trophectoderm (TE; purple dotted lines; late TE versus EPI log2FC [fold-change] > 2, n = 409) or in primed hPSCs (red dotted lines; primed versus naive; Stirparo et al., 2018; log2FC > 2, n = 1,778). (B) One-way hierarchical clustering of differentially expressed genes between late TE (blue) and epiblast (EPI; red) (top 200 up- and down-ranked genes; rank is the product of –log10padj [adjusted p-value] and FC) in HNES1 time courses under the indicated conditions. (C) Log2 FPKM (fragments per kilobase per million mapped reads) expression value for selected TE genes under A83, N2B27, PD, and PD+A83 conditions. Error bars are from biological duplicates. (D) Heatmap of *Z* score centered values for clusters identified in the PD+A83 time course (cluster 1, 1,787; cluster 2, 2,594; cluster 3, 1,872; cluster 4, 1,227; cluster 5, 2,055; cluster 6, 1,839). (E) Ratio of modulated genes between eTE/ICM (blue and green) and lTE/EPI (purple and red) in each cluster. (F) Bootstrap Spearman correlation (100 iterations, number of genes = 50) between the PD+A83 time course (PXGL, d1,d2,d3,d5, log2 expression > 1) and human embryo stages.

We carried out whole-transcriptome sequencing over a 5-day time course of naive cell differentiation in N2B27 alone or with PD, A83, or PD+A83. Libraries were prepared in duplicate from embryo-derived HNES1 and reset cR-H9 cells. Principal-component analysis (PCA) aligned samples according to treatment and time along two distinct trajectories (Figure 3A). N2B27 and A83 cultures followed the formative capacitation pathway, culminating in the region of density overlay for genes upregulated in conventional hPSCs. PD and PD+A83 samples followed an alternative path, extending to the high-density area for genes upregulated in late trophectoderm in the human blastocyst (Petropoulos et al., 2016; Figures S3A–S3E). Hierarchical clustering using differentially expressed genes in the embryo substantiated conversion in PD or PD+A83 into a population with trophectoderm features (Figures 3B and S3F). Trophectoderm genes were not upregulated in N2B27 or A83 alone. Figure 3C shows examples of expression dynamics *in vitro* and in the embryo. Upregulation of *GATA2*, *CDX2*, and *TBX3* began from day 1 and other markers from day 2 or day 3. Some genes (*TFAP2C*, *TBX3*, and *HAVCR1*) prominent in trophectoderm also showed appreciable expression in naive hPSCs (Figure 3C) but were upregulated further in PD and PD+A83.

For independent comparison with primate embryo development, we used transcriptome data from *Macaca fascicularis* (Nakamura et al., 2016). We averaged the scRNA-seq (single-cell RNA-seq) embryo data according to developmental tissue and stage and computed the integrated PCA with orthologous genes. The N2B27 and A83 time course gained similarity to post-implantation epiblast, whereas the PD and PD+A83 trajectories related to trophectoderm formation (Figure S3G).

In PD+A83, we identified 6 clusters of dynamically expressed genes (Figure 3D). For each cluster, we determined relative representation of profiles of early and late trophectoderm, ICM, and pre-implantation epiblast from the embryo. Clusters 3–6 displayed increasing relationships to early trophectoderm compared with ICM and to late trophectoderm compared with epiblast (Figure 3E). We also compared each day of the time course with the embryo samples. Bootstrap Spearman analysis showed an increasing correlation with early trophectoderm from day 1 and with late trophectoderm on day 5. Epiblast correlation declined on day 5 (Figure 3F). Correlation with ICM remained low (<0.7) at all time points.

These transcriptome analyses show that human naive stem cells in PD+A83 do not undergo formative transition but differentiate into trophectoderm via a separate and direct path.

### Single-cell transcriptome analysis of differentiation trajectory

**Figure 4:**
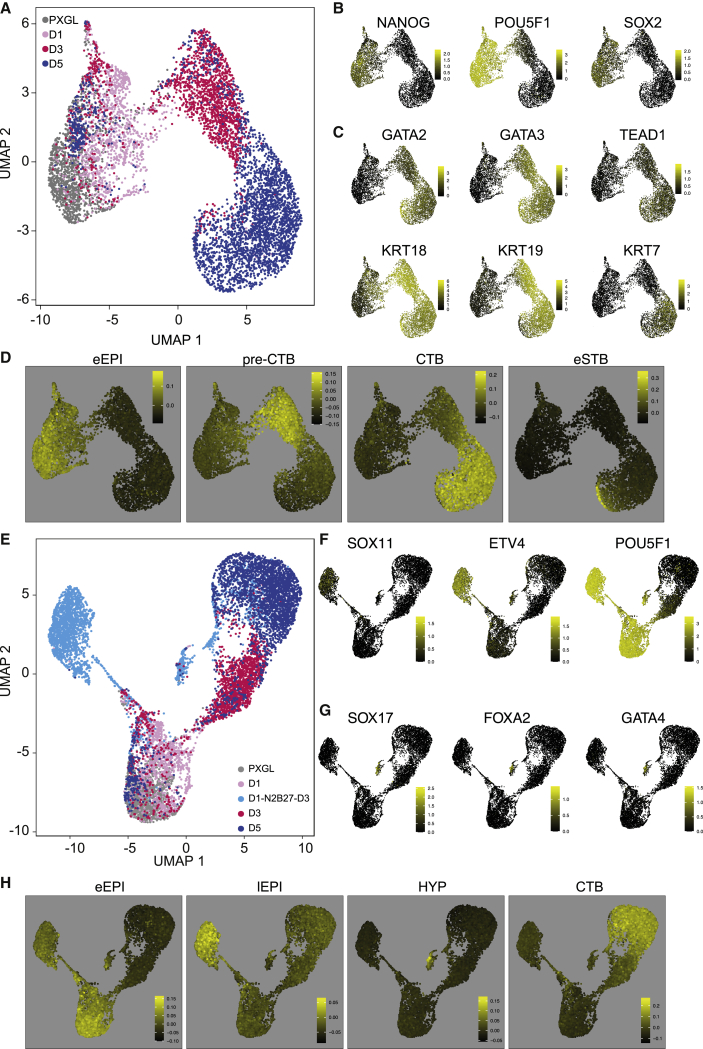
Single-cell analysis (A) UMAP of the PD+A83 time course, colored according to sample day. (B) Expression of selected pluripotency markers in (A). (C) Expression of selected TE and early trophoblast markers in (A). (D) Expression in (A) of genes enriched in the indicated human embryo stages (Xiang et al., 2019): eEPI, E6–E8 Epi; preCTB, TE (E6–E7); CTB, cytotrophoblast; eSTB, early syncytiotrophoblast. (E) UMAP with addition of cells cultured for 24 h in PD+A83 followed by 3 days in N2B27 only. (F) Expression of selected post-implantation Epi markers in (E). (G) Expression of selected hypoblast markers in (E). (H) Expression in (E) of genes enriched in the indicated human embryo stages (Xiang et al., 2019): eEPI, E6–E8 Epi; mEPI, E9–E10 Epi; lEPI, E12–E14 Epi; HYP, hypoblast.

To obtain higher resolution of the differentiation trajectory we performed single-cell transcriptome analysis using the 10X Genomics platform. We prepared samples on days 0, 1, 3, and 5 of the PD+A83 time course. A total of 14,396 cells passed quality control, with more than 3,000 genes detected. UMAP (uniform manifold approximation and projection) visualization showed a relatively continuous and synchronous progression (Figure 4A). Downregulation of pluripotency markers was reciprocal to upregulation of trophectoderm genes in the vast majority of cells on days 3 and 5 (Figures 4B and 4C). A minor fraction of day 5 cells expressed naive factors and clustered with the day 1 population. Significantly, no cells from day 0 clustered with day 3 or day 5 cells, indicating that trophectoderm differentiation does not pre-exist under PXGL culture conditions.

We utilized data from extended cultures of human embryos (Xiang et al., 2019) to define gene signatures for tissues and stages, including post-implantation trophoblast types. Computing the distribution of tissue-specific profiles on the UMAP showed conversion from naive epiblast similarity on day 0 to trophectoderm (called preCTB by Xiang et al., 2019) by day 3 and cytotrophoblast on day 5 (Figure 4D). We also saw that a subset of day 5 cells exhibited features of early syncytiotrophoblast. Inspection of selected trophectoderm and trophoblast marker genes substantiated these relationships (Figures S4A and S4B), consistent with immunostaining (Figure 2A)

We also examined the fate of cultures treated with PD+A83 for only 24 h and then released into N2B27 for 3 days. Integration of this sample (5,030 cells) into the UMAP did not affect the major clusters of trophectoderm and cytotrohoblast. However, relatively few of these cells progressed to trophectoderm (Figure 4E). Instead, they mainly populated a new cluster that lacked naive pluripotency factors but expressed general pluripotency and post-implantation epiblast markers (Figure 4F). Interestingly, a stream of cells connecting this cluster with naive and day 1 cells expressed genes enriched in embryonic day 8 (E8)–E11 epiblast (Figure S4C), consistent with progression via formative mid-epiblast toward primed late epiblast. A second smaller cluster exhibited a repertoire of hypoblast marker genes (Figures 4G and S4D). A few cells from day 3 and day 5 of PD+A83 treatment co-located in this cluster. Distribution of embryo tissue profiles substantiated post-implantation epiblast and hypoblast assignations (Figure 4H).

We immunostained cultures treated with PD+A83 for 24 h and then for 2 days with A83 alone or N2B27. In both cases, we saw exclusive expression of GATA3, OCT4, or the hypoblast marker SOX17 (Figure S4E). Notably, however, the GATA3 population was predominant in A83, whereas the majority of cells were OCT4-positive in N2B27. We surmise that cells treated with PD+A83 for 24 h are mostly still flexible in fate choice and can become trophectoderm, hypoblast, or formative epiblast.

### Genetic perturbation of regulatory transcription factors

In mouse ESCs, deletion of *Oct4* or *Sox2* results in upregulation of *Cdx2* and differentiation to trophoblast-like cells. In contrast, deletion of *Nanog* provokes differentiation to hypoblast-like cells with no evidence of trophectoderm (Mitsui et al., 2003). To determine whether these relationships are conserved in human naive cells, we performed targeted mutagenesis using two different CRISPR-Cas9 methodologies.

**Figure 5:**
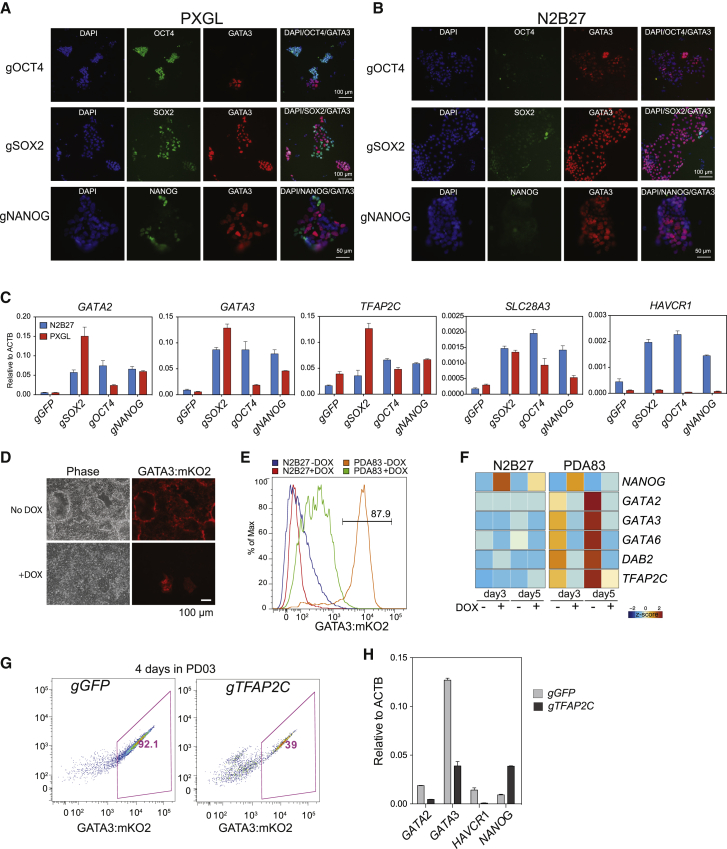
Genetic perturbations (A) Immunostaining for the indicated markers after Cas9/gRNA RNP targeting of *OCT4*, *SOX2*, or *NANOG* in HNES1 cells and maintenance in PXGL for 5 days. (B) As (A), but the culture was changed to N2B27 after 1 day. (C) qRT-PCR assay of TE marker expression after Cas9/gRNA RNP targeting of the indicated genes in HNES1 cells. Error bars from technical duplicates. (D) GATA3:mKO2 cells with or without DOX (doxycline) induction of *NANOG* in PD+A83 for 5 days. (E) Flow cytometry histogram of GATA3:mKO2 expression in N2B27 or PD+A83 with or without DOX induction of *NANOG*. (F) Heatmap of qRT-PCR gene expression values with and without DOX induction of NANOG in N2B27 or PD+A83. (G) GATA3:mKO2 flow cytometry plots after *GFP* (control) or *TFAP2C* targeting by gRNA plasmid transfection and culture for 4 days in PD. (H) qRT-PCR assay of marker expression after *GFP* or *TFAP2C* targeting and culture as in (H). Error bars from technical duplicates.

We first mutated *OCT4*, *SOX2*, and *NANOG* in parental HNES1 cells by transfection with Cas9 and gRNA (guide RNA) ribonucleoprotein (RNP) complexes. Consistent with their expected essential roles, we observed reduced naive colony numbers in PXGL for all three genes (Figure S5A). To assess the fate of targeted cells, we performed immunostaining for GATA3 together with the targeted transcription factor on cells maintained in PXGL or exchanged into N2B27 after 24 h. On day 5 after transfection, clusters of GATA3-positive cells were apparent in each case (Figures 5A and 5B). In PXGL, depletion of the targeted transcription factor was evident in the cells that were GATA3 positive. qRT-PCR confirmed upregulation of trophectoderm markers (Figure 5C).

RNP transfection efficiency can limit mutation frequency. We therefore introduced a constitutive Cas9 expression construct into the *AAVS1* locus in naive GATA3:mKO2 reporter cells. We then used Piggybac (PB) transposition to integrate gRNA expression constructs containing a selectable marker. After transfection and selection, there was a massive reduction in colony numbers in PXGL for all three knockouts (Figure S5B). We saw activation of the mKO2 reporter in N2B27 with or without A83 (Figure S5C). Notably, the *SOX2* knockout had a pronounced phenotype, with around 50% and 90% of cells positive for mKO2 in N2B27 and A83, respectively (Figure S5C).

Trophectoderm differentiation in response to *NANOG* targeting is at variance with the phenotype in mouse ESCs, suggesting a function specific to human naive cells. This prompted us to investigate whether NANOG can suppress trophectoderm formation. We introduced a doxycycline-inducible *NANOG* expression vector into GATA3:mKO2-naive cells. Induction of NANOG prevented the appearance of mKO2-positive cells (Figures 5D and 5E) and suppressed upregulation of trophectoderm markers in PD+A83 (Figure 5F).

Substantial expression of TFAP2C is a distinctive feature of human naive stem cells and pre-implantation epiblast cells (Boroviak et al., 2018; Pastor et al., 2018; Stirparo et al., 2018). In the mouse, TFAP2C is known as a trophoblast factor (Cao et al., 2015; Choi et al., 2012). It is barely expressed in mouse ICM, early epiblast, or ESCs, and forced expression provokes trophoblast-like differentiation (Adachi et al., 2013; Kuckenberg et al., 2010). We targeted *TFAP2C* in Cas9-expressing cells. We saw a reduction in colony numbers in PXGL (Figure S5B), consistent with a report that TFAP2C may be required for stable propagation of human naive cells (Pastor et al., 2018). In contrast to the other transcription factors, however, mKO2 was not elevated. TFAP2C knockout populations showed reduced production of mKO2-high cells in PD (Figure 5G) and lower expression of trophectoderm markers (Figure 5H). We obtained similar results with the Cas9/gRNA RNP method (Figure S5D).

These observations indicate that OCT4, SOX2, and NANOG suppress trophectoderm, whereas TFAP2C has dual effects, supporting naive stem cell self-renewal but also enabling trophectoderm differentiation.

### Differentiation of post-implantation-stage hPSCs

There are contested reports that BMP induces conventional hPSCs to form placental trophoblast-like cells (Amita et al., 2013; Bernardo et al., 2011; Lee et al., 2016; Roberts et al., 2014; Xu et al., 2002; Yabe et al., 2016). We investigated whether BMP signaling was required for trophectoderm induction from naive cells. We found that addition of BMP or the BMP receptor inhibitor LDN-193189 (LDN) had a negligible effect on induction of GATA3:mKO2 in PD+A83 (Figure S6A). Furthermore, BMP signaling is much lower in naive cells compared with primed hPSCs (Figure S6B).

**Figure 6:**
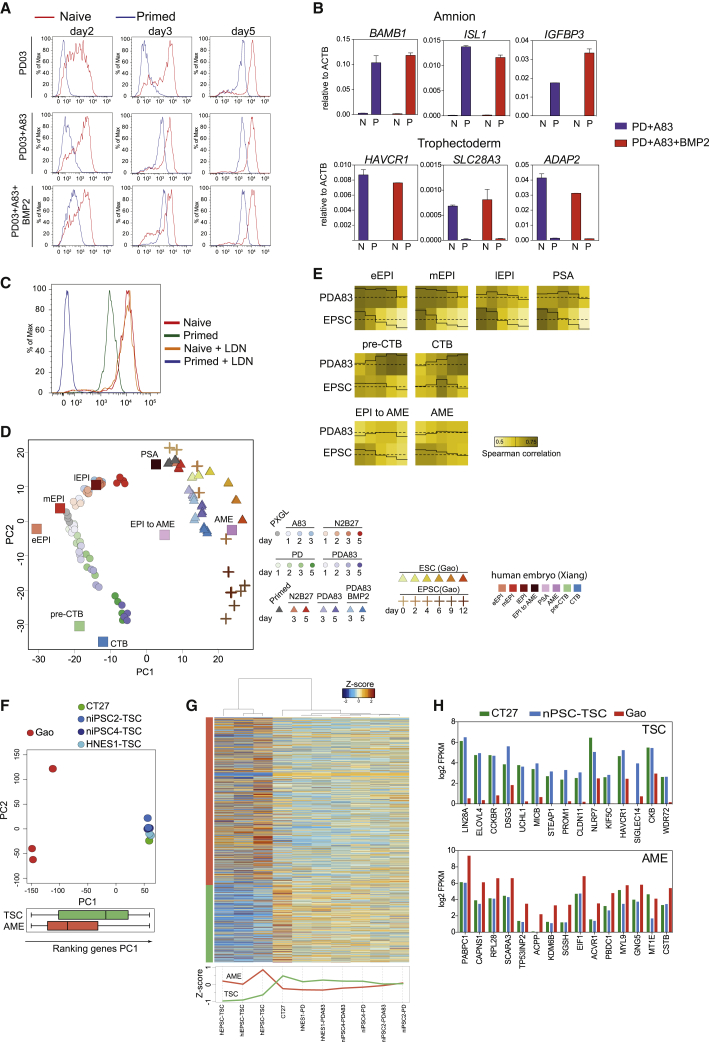
Potency of naive versus primed hPSCs (A) Flow cytometry analysis of naive and primed GATA3:mKO2 cells in PD03, PD03+A83, or PD03+A83+BMP2. (B) qRT-PCR assay of selected AME and TE markers after 5 days culture of naive (N) or primed (P) cells in PD03+A83 with or without BMP2. Error bars from technical duplicates. (C) Flow cytometry analysis of naive and primed GATA3:mKO2 cells in PD03+A83 with or without the BMP inhibitor LDN. (D) PCA of RNAseq data fromdifferentiation time courses for naive cells (HNES1 and cR-H9), hEPSCs (Gao et al., 2019), and primed hPSCs (H9, HNES1), together with averaged values for human embryo stages during extended *in vitro* development (Xiang et al., 2019). Computed using the 1,000 most variable genes between embryo stages with log_2_FPKM ≥ 1 in at least one stage. (E) Traced heatmap computed with median of bootstrap Spearman correlation (iteration 100, number of genes = 50). (F) Top: PCA computed with all expressed genes for hEPSC derivatives (Gao et al., 2019), placental TSCs (CT27), and N stem-cell-derived TSC samples. Values from this study are averages from biological duplicates. Naive stem-cell-derived TSCs were generated after initial induction with PD only or with PD+A83. Bottom: Box and whisker plot of distribution along PC1 of genes enriched for expression in TSCs (Okae et al., 2018) compared with trophoblast lineages in the embryo or in AME compared with other embryo stages (Xiang et al., 2019). (G) Top: heatmap computed with AME- and TSC-enriched genes for hEPSC-derived cells, placental cytotrophoblast TSCs, and naive cell-derived TSCs. Bottom: median *Z* score for AME- and TSC-enriched genes in cell line samples. (H) Log2 FPKM-averaged expression in cells from the indicated studies of the top 15 differentially enriched genes in TSCs or AME.

To compare differentiation behaviors in an isogenic setting, we converted GATA3:mKO2 cells to conventional hPSC status (Rostovskaya et al., 2019). We then assayed induction of mKO2 in response to PD, PD+A83, or PD+A83+BMP (Figure 6A). For the converted cells, reporter expression was negligible on day 2 under any condition. mKO2-positive cells appeared by day 3 in PD+A83 or by day 5 in PD. BMP accelerated these kinetics. However, reporter levels were at least 10-fold lower relative to differentiation from naive counterparts. We also noted that A83 or BMP alone induced low expression of mKO2 in a fraction of converted cells but had no effect on naive cells (Figure S6C). qRT-PCR analysis confirmed log-fold lower upregulation of GATA3 in conventional compared with naive hPSCs (Figure S6D).

GATA3 is also expressed in amnion, and recently it has been reported that conventional hPSCs differentiate into amnion-like cells in response to BMP (Zheng et al., 2019). In conventional but not naive cell differentiation, we detected upregulation of markers reported by Zheng et al. (2019) that are also present in early amnion of cultured human embryos (Xiang et al., 2019; Figure 6B). BMP inhibition with LDN blocked expression of GATA3 and of amnion markers in conventional hPSCs (Figures 6C and S6D) and steered differentiation into the neural lineage (Figure S6E).

hEPSCs have been reported to produce trophoblast in response to A83 and BMP (Gao et al., 2019). We sought to clarify the relationship between naive and conventional hPSC or hEPSC lineage trajectories. We examined the transcriptomes of undifferentiated hEPSCs (Gao et al., 2019; Yang et al., 2017) and confirmed that they are distinct from naive stem cells and similar to conventional hPSCs (Stirparo et al., 2018; Figure S6F). We compared naive and conventional hPSCs and hEPSCs with epiblast stages in the human embryo (Xiang et al., 2019). PCA using differentially expressed genes in the embryo (Figure S6G) corroborated the close relationship between naive stem cells propagated in PXGL and pre-implantation epiblast (Bredenkamp et al., 2019b; Stirparo et al., 2018), whereas hEPSCs and conventional hPSCs are related to post-implantation epiblast from day 10 onward.

We analyzed collated transcriptomes from hPSC, hEPSC (Gao et al., 2019), and naive cell differentiation time courses in comparison with human embryo extended culture samples (Xiang et al., 2019), which included post-implantation epiblast-to-amnion transition and amnion (AME). PCA resolved distinct trajectories for naive cells compared with conventional hPSCs and hEPSCs (Figure 6D). Naive cell differentiation in PD+A83 proceeded via similarity to trophectoderm (called preCTB by Xiang et al., 2019) and culminated in proximity to cytotrophoblast. In contrast, conventional hPSC and hEPSC differentiation was related to early AME, with hEPSCs proceeding further. We extracted genes with enriched expression in AME-like cells (Zheng et al., 2019) and examined their distribution in the PCA. The intensity overlay was concentrated in the area occupied by the endpoint of hEPSC differentiation (Figure S6H). Analysis using AME samples from *Macaca* embryo cultures (Ma et al., 2019) produced a similar outcome, with the intensity distribution concentrated in the region of differentiated hEPSCs (Figure S6I). We used bootstrap Spearman analysis to examine global correlation between embryo stages and *in vitro* naive or hEPSC differentiation. The traced heatmaps show that naive cell differentiation progressed from high starting correlation with pre-implantation epiblast to similarity with trophectoderm (preCTB) and cytotrophoblast (Figure 6E). In contrast, hEPSCs lost high initial relatedness to post-implantation epiblast but did not gain correlation with trophectoderm.

hEPSCs were reported to give rise to TSCs by direct transfer to TSC culture medium (Gao et al., 2019). We compared the transcriptome of hEPSC derivatives (Gao et al., 2019) with the placental cytotrophoblast-derived TSC line CT27 (Okae et al., 2018) cultured in our laboratory and TSCs derived from naive cells after induction with PD only or PD+A83. PCA computed with all protein-coding genes shows naive stem-cell-derived and placental TSCs clustered together but well separated from hEPSC progeny on PC1 (Figure 6F). TSC and AME-enriched genes were distributed differentially along PC1. TSC-enriched genes were more highly represented in naive stem cell and placenta-derived TSCs, whereas hEPSC-derived cells showed higher expression of AME-enriched genes, although many of these were also detected in TSCs (Figures S6J and S6K). Hierarchical clustering substantiated this finding (Figure 6G). Inspection of the top differentially expressed genes confirmed that TSCs generated from naive cells expressed TSC markers at levels comparable with placental TSCs and much higher than EPSC derivatives (Figure 6H). Conversely, AME markers were expressed more highly in differentiated EPSCs.

We also examined published transcriptome data for differentiation of conventional hPSCs induced with a combination of BMP, A83, and fibroblast growth factor receptor (FGFR) inhibition (BAP) (Yabe et al., 2016). Bootstrap Spearman correlation analysis showed no significant relationship to naive cell differentiation in PD+A83 but high correlation with days 4–9 of hEPSC differentiation (Figure S6L). Several AME-enriched genes were expressed in BAP cells similarly as EPSC derivatives (Figure S6M).

These analyses demonstrate that naive and non-naive hPSCs differentiate along BMP-independent and BMP-dependent trajectories, respectively, into distinct trophectoderm or AME-like fates.

### Epiblast in the human blastocyst retains trophectoderm lineage plasticity

**Figure 7:**
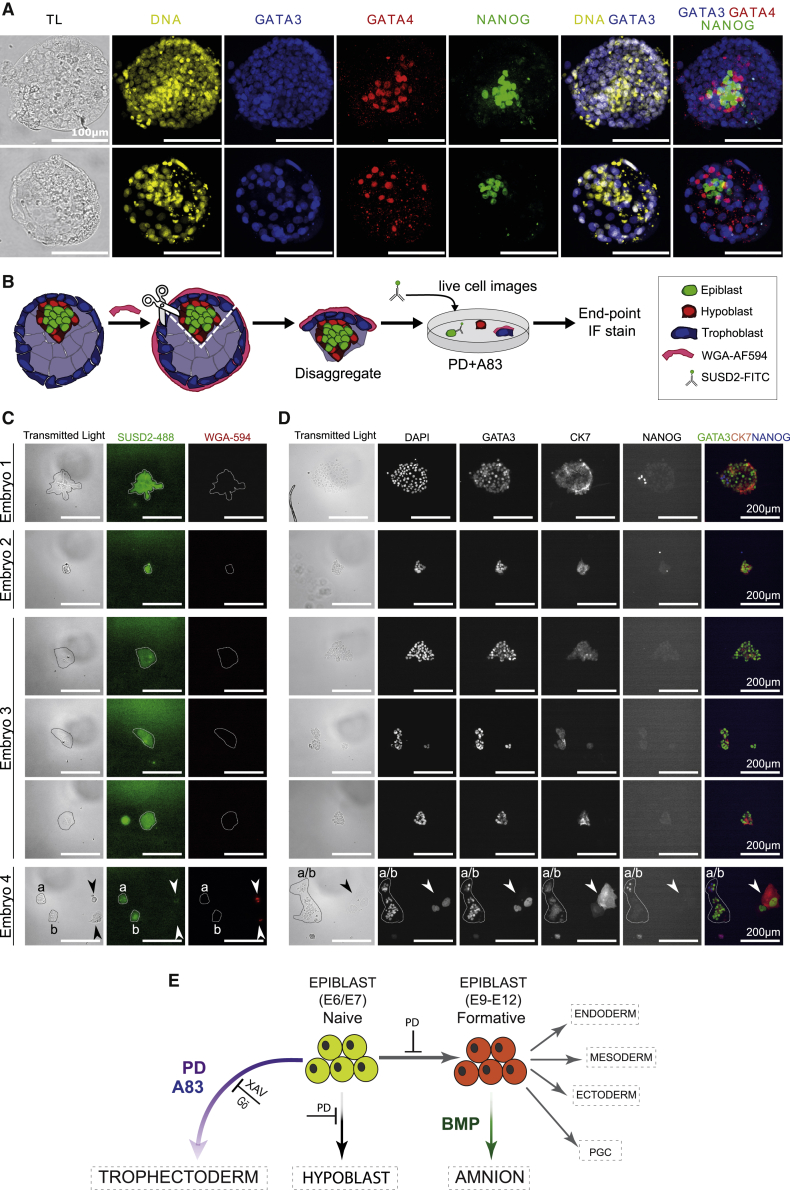
Plasticity of human embryo pre-implantation Epi (A) Immunostaining of fully expanded human blastocysts as used in this study for markers of TE, Epi, and HYP. (B) Schematic of live-cell labeling of TE and Epi cells for culture analysis. (C) Live-cell images after SUSD2 labeling 20 h after plating. In the bottom panel, a and b denote separate SUSD2-positive cell clusters, and arrows indicate SUSD2-negative, WGA-positive cells. (D) Immunostaining of cultures in (D) after culture for 96 h. (E) Schematic summary of findings showing that N cells can differentiate to TE or progress to formative pluripotency with a switch of lineage competence from TE to AME.

*Ex vivo* culture conditions may corrupt cell identities or alter developmental potential. We therefore examined whether the capacity of naive stem cells to generate trophectoderm is an authentic feature of epiblast cells in late human blastocysts (E6 and E7) in which hypoblast is already specified (Niakan and Eggan, 2013; Roode et al., 2012). Frozen blastocysts (E5 or E6) were thawed and cultured for 24 h for formation of fully expanded blastocysts, some of which commenced hatching (Figure S7A). Immunostaining confirmed that GATA3 was confined to trophectoderm cells and that GATA4-positive hypoblast had segregated (Figure 7A). We isolated ICMs for explant culture by immunosurgical removal of trophectoderm (Solter and Knowles, 1975). ICMs were maintained intact and plated on dishes coated with laminin 111-E8 (Kiyozumi et al., 2020) in N2B27 with or without PD+A83. In N2B27, a central mass of relatively undifferentiated cells persisted in most cases, but patches of trophectoderm morphology often outgrew. Immunostaining after 5 days showed expression of NANOG in undifferentiated cell masses and of GATA3, CK7, and hCGB in peripheral cells (Figure S7B). In contrast, in PD+A83, explants invariably differentiated almost entirely into trophectoderm and trophoblast cells. GATA3 was expressed throughout the explants together with patches of CK7- and hCGB-positive cells by day 5. The BMP inhibitor LDN did not impede outgrowth of GATA3-positive cells (Figure S7C; Video S3).

Video S3. Time lapse of immunosurgically isolated ICM explant in PD+A83 with LDN for 48 h, Related to Figure 7

These observations suggest that epiblasts from expanded human blastocysts can regenerate trophectoderm. However, the possibility that polar trophectoderm cells may persist after immunosurgery cannot be excluded. We therefore adopted an alternative approach (Figure 7B). We removed the mural trophectoderm by microdissection and plated the ICM/polar trophectoderm composites. After 16 h, we performed live-cell immunostaining for the epiblast marker SUSD2 (Bredenkamp et al., 2019a). The antibody labeled ICM cells but not peripheral trophoblast (Figure S7D). Daily imaging showed morphological changes with no detachment or appreciable death of ICM cells. After 5 days in PD+A83, the explant almost entirely comprised GATA3- and CK7-positive cells (Figure S7E).

Finally, to confirm epiblast-to-trophectoderm conversion, we incubated intact blastocysts with conjugated wheat germ agglutinin (WGA), labeling all outside cells prior to microdissection (De Paepe et al., 2013; Figures 7B and S7F). Dissected WGA-labeled embryo fragments were dissociated using Accutase and plated in PD+A83 with addition of Rho-associated kinase inhibitor to maximize cell viability. After 20 h, we performed live-cell staining for SUSD2 and captured images, registering WGA+SUSD2-negative (trophectoderm) and WGA-SUSD2+ (epiblast) cell clusters (Figure 7C). Cultures were maintained for 4 days in PD+A83 before fixation and immunostaining. Figure 7D shows tracked WGA-negative SUSD2-positive clusters from 4 embryos, all of which gave rise to GATA3-positive cells. CK7 staining could also be detected among the GATA3-positive cells, although weaker than in colonies formed from WGA-positive SUSD2-negative clusters corresponding to pre-formed trophectoderm.

These findings confirm that human epiblast cells retain plasticity to form trophectoderm. They support a model of pluripotent lineage progression in which trophectoderm potency in humans is retained until the formative transition to competence for AME, germline, and germ layers (Figure 7E). Interestingly, this sweeping change in developmental capacity appears coincident with gain in BMP responsiveness.

## Discussion

Formation of trophectoderm is the first differentiation event in mammalian embryogenesis. In the mouse, fate mapping and molecular studies have established a strict lineage bifurcation so that, when the blastocyst forms, ICM cells become refractory to further trophectoderm specification. Our findings expose a different scenario in humans, where trophectoderm lineage potential is maintained as the blastocyst matures. Human naive epiblast stem cells can form trophectoderm with high efficiency, and epiblast cells extracted from late human blastocysts robustly regenerate trophectoderm. Lineage restriction is imposed upon formative transition. Although post-implantation-stage hPSCs can produce epithelial cells that are superficially similar to trophoblast, global profiling indicates a higher resemblance to AME, the first lineage to segregate from the embryonic disk in primate embryos (Boroviak and Nichols, 2017). These findings resolve contradictory reports and re-establish a lineage hierarchy consistent with *in vivo* development. More broadly, our results identify developmental plasticity in the human naive epiblast that is not precedented by studies in the mouse and is likely to confer higher regulative capacity in the human embryo and potentially other mammals.

Human naive stem cells are generated and stably maintained in PXGL medium (Bredenkamp et al., 2019b; Guo et al., 2017), which comprises three small-molecule inhibitors of MEK/ERK, Wnt, and aPKC (atypical protein kinase C) signaling, respectively. These requirements, which differ from those for mouse ESC propagation (Dunn et al., 2014; Ying et al., 2008), can now be better understood. In both species, MEK/ERK inhibition prevents formative pluripotency transition to post-implantation epiblast (Smith, 2017). In humans, however, PD also promotes differentiation from naive epiblast to trophectoderm. Inhibition of Wnt and aPKC block access to trophectoderm, consolidating self-renewal. Thus, triple blockade of signaling input into the gene-regulatory network is required to constrain cells in the naive epiblast state. In addition, autocrine activation of SMAD2 and SMAD3 by Nodal contributes to suppression of trophectoderm. Notably, Nodal and GDF3 are highly expressed in naive epiblast in the human embryo (Blakeley et al., 2015; Stirparo et al., 2018). In other species, however, exogenous stimulation may facilitate naive stem cell propagation.

Two recent papers have reported derivation of TSCs from human naive stem cells by selective amplification of relatively rare cells (Cinkornpumin et al., 2020; Dong et al., 2020). However, it is unclear from those studies how the TSCs arose. In contrast, our findings establish a robust developmental trajectory from naive epiblast to trophectoderm and, thence, cytotrophoblast and other trophoblast lineages. We also show that TSCs derived via trophectoderm induction are transcriptomically similar to placental TSCs. Our findings are corroborated in a parallel study by Io et al. (2021).

Deletions of core pluripotency factors reveal common and species-specific features. *OCT4* and *SOX2* knockouts trigger trophoblast formation from naive stem cells in mice and humans. *NANOG* deletion also releases trophectoderm differentiation in human naive cells, whereas mutation in mouse ESCs predisposes to hypoblast (Chambers et al., 2007; Mitsui et al., 2003). NANOG may have a conserved function to establish and consolidate naive epiblast identity, but the outcome of mutation differs because of the lack of trophectoderm restriction in humans. Requirement for TFAP2C as a mediator of trophectoderm formation (Cao et al., 2015; Choi et al., 2012) appears to be conserved. However, in humans, TFAP2C is also expressed in ICM and epiblast and plays a role in maintenance of naive stem cell self-renewal (Pastor et al., 2018). We speculate that the dual functionality of TFAP2C may be central to the lineage plasticity of human naive cells.

There have been several reports that conventional hPSCs and hEPSCs can differentiate into epithelial cells that exhibit some markers of trophectoderm (Amita et al., 2013; Gao et al., 2019; Xu et al., 2002; Yang et al., 2017). However, global transcriptome profiling indicates a trajectory unrelated to pre-implantation development. Instead, differentiation proceeds toward a post-implantation extraembryonic lineage, AME. Significantly, BMP signaling is essential for AME-like differentiation from primed stem cells, whereas trophectoderm induction from naive cells is insensitive to this pathway. BMP independence is consistent with the lack of a BMP4 effect on trophectoderm formation in human embryo culture (De Paepe et al., 2019). Furthermore, naive cells show weak SMAD activation when exposed to BMP, and responsiveness to this pathway increases after formative transition.

Our findings demonstrate that human ICM explants from E6 blastocysts have unprecedented plasticity to reform trophectoderm. Mouse ICM loses trophectoderm potency entirely by the mid-blastocyst stage, before epiblast specification (Posfai et al., 2017). By E6, the human ICM has already segregated into hypoblast and epiblast (Niakan and Eggan, 2013; Petropoulos et al., 2016; Roode et al., 2012; Stirparo et al., 2018; Xiang et al., 2019). We saw that patches of trophectoderm grow out from human ICMs in N2B27 alone. This could be attributable to persistence of some polar trophectoderm cells after immunosurgery. However, treatment with PD+A83 caused conversion of almost the entire ICM into trophectoderm and differentiated trophoblast. The ICM origin of regenerated trophectoderm under these conditions was confirmed by prior live-cell staining. This finding raises the intriguing possibility that epiblast may contribute continuously to the normal expansion of trophectoderm in the late blastocyst. Alternatively, epiblast plasticity may be reserved for reconstitution after cell loss.

Our results establish that the mouse paradigm of early lineage segregation is not adhered to in humans and that human naive cells have intrinsic potential for trophectoderm formation. Interestingly, it has been reported that human trophectoderm at E5 can regenerate an ICM population (De Paepe et al., 2013). High regulative flexibility may be an important mechanism for safeguarding human embryos. In the context of assisted conception, this could explain why viable pregnancies can ensue from embryos that are judged morphologically to be of lower quality or incur cell damage during blastocyst freezing and thawing. We speculate that retained trophectoderm potency may be a more widespread feature of early mammalian embryology that has eroded in rodents, associated with their early implantation and rapid development. Suppressing trophectoderm differentiation may be a common requirement for propagation of naive pluripotent stem cells.

Recently, advances in human embryo culture have been reported (Deglincerti et al., 2016; Shahbazi et al., 2016; Xiang et al., 2019). However, availability of human embryos is limited, and quality is variable. Our study illustrates that human naive stem cells are a complementary, experimentally convenient, model for delineating the molecular mechanisms of early lineage segregation and uncovering species-specific features. Considerable current interest also focuses on production of stem-cell-based blastocyst models, achieved by combining mouse ESCs and TSCs (Hyun et al., 2020; Rivron et al., 2018). We suggest that competency to produce all three primary lineages may enable constitution solely from human naive stem cells of a blastocyst entity with full developmental potential. With the additional advantage of efficient clonal genome engineering, this would be an attractive system for elucidating principles of embryo self-organization.

### Limitations of study

The limited numbers of human embryos available for research and their variable quality are major challenges. Our observations are reproducible over multiple experiments but each with only a small number of ICMs. With a more consistent supply of human embryos, it could be feasible to implement a live-cell tracking approach in intact embryos to investigate whether ICM and epiblast cells contribute continuously to trophectoderm in the unperturbed blastocyst or only in a regenerative context. Furthermore, there are very few transcriptome datasets available for cultures of human embryos to early post-implantation stages. Comparison with additional data is needed to definitively establish the identity of differentiated cells induced by BMP-based treatments of conventional or hEPSCs. Although our findings show resemblance to AME, it cannot be ruled out that, under certain culture conditions, trophoblast-like cells may arise by transdifferentiation.

## STAR★Methods

### Key resources table

**Table:** 

REAGENT or RESOURCE	SOURCE	IDENTIFIER
**Antibodies/target**		

SOX2	Santacruz	Cat#sc-365823; RRID:AB_10842165
NANOG	R&D	Cat#AF1997; RRID:AB_355097
OCT4(C-10)	Santa Cruz	Cat#sc-5279; RRID:AB_628051
GATA3	Abcam	Cat#ab199428; RRID:AB_2819013
KLF17	Atlas Antibody	Cat#HPA024629; RRID:AB_1668927
SOX17	R&D	Cat#AF1924; RRID:AB_355060
FOXA2	R&D	Cat#AF2400; RRID:AB_2294104
CK7	Abcam	Cat#AB181598; RRID:AB_2783822
CK18	Abcam	Cat#AB133263; RRID:AB_11155892
HLA-G	Abcam	Cat#AB52455; RRID:AB_880552
hCGB	Abcam	Cat#AB9582; RRID:AB_296507
PKCi	Novus Biologicals	Cat#NBP1-84959; RRID:AB_11033145
PAR6B	Santa Cruz	Cat#sc-166405; RRID:AB_2267890
AQP3	Abcam	Cat#AB153694
SUSD2 Antibody, anti-human, VioBright FITC	Miltenyi Biotech	Cat#130-106-401; RRID:AB_2653618
pSMAD 2	Cell Signaling	Cat#3101; RRID:AB_331673
pSMAD 1/5/9	Cell Signaling	Cat#9511; RRID:AB_331671
beta tubulin	Abcam	Cat#AB6046;RRID:AB_2210370

**Chemicals, peptides, and recombinant proteins**		

MEK inhibitor PD0325901	ABCR	Cat#AB 253775
GSK3 inhibitor CHIR99021	ABCR	Cat#AB 253776
Tankyrase inhibitor XAV939	Cell Guidance Systems	Cat#SMS38-200
aPKC inhibitor Gö6983	Bio-Techne	Cat#2285
ROCK inhibitor Y-27632	Merck Chemicals	Cat#688000-100MG
LIF	Made in-house	N/A
Activin A	Made in-house	N/A
Fgf2	Made in-house	N/A
BMP2	Made in-house	N/A
Activin receptor inhibitor A83-01	Generon	Cat#A12358-50
BMP receptor inhibitor LDN-193189	Axon Medchem	Cat#Axon 1509
TrueCut Cas9 Protein v2	ThermoFisher Scientific	Cat#A36498
Phalloidin, Alexa Fluor 555 conjugate	Cell Signal	Cat#8953S
Wheat Germ Agglutinin, Alexa Fluor 594 Conjugate	Thermo Fisher Scientific	Cat#W11262

**Complete culture media and cell dissociation reagent**		

N2B27	Made in-house	N/A
AFX	Made in-house	N/A
E8	Made in-house	N/A
mTeSR1	StemCell Technologies, Inc.	Cat#05850
Accutase	Millipore	Cat#SCR005
TrypLE Express Enzyme	Thermo Fisher Scientific	Cat#12605028

**Cell attachment proteins and peptides**		

Geltrex	Thermo Fisher Scientific	Cat#A1413302
Laminin	Millipore	Cat#CC095-5MG
Fibronectin	Millipore	Cat#FC010
Laminin 111-E8	From Kiyotoshi Sekiguchi	N/A

**Critical commercial kits**		

Alkaline Phosphatase Kit	Sigma-Aldrich	Cat#86R-1KT
Neon 10ul transfection kit	Thermo Fisher Scientific	Cat#MPK1096
Neon 100ul transfection kit	Thermo Fisher Scientific	Cat#MPK10096

**Deposited data**		

RNaseq	This study	GEO: GSE166401
RNaseq	This study	GEO: GSE167089
RNaseq	Yabe et al., 2016	GEO: GSE73017
RNaseq	Gao et al., 2019	E-MTAB-7253
scRNaseq	This study	GEO: GSE166422
scRNaseq	Petropoulos et al., 2016	E-MTAB-3929
scRNaseq	Nakamura et al., 2016	GEO: GSE74767
scRNaseq	Ma et al., 2019	GEO: GSE130114
scRNaseq	Xiang et al., 2019	GEO: GSE136447

**Experimental models: cell lines**		

HNES1	Guo et al., 2016	N/A
HNES1_GATA3mKO2	This study	N/A
cR-H9	Guo et al., 2017	N/A
cR-NCRM2	Guo et al., 2017	N/A
cR-Shef6	Guo et al., 2017	N/A
H9	WiCell	WA09
CT27	Okae et al., 2018	N/A

**Oligonucleotides**		

TrueGuide tracrRNA	ThermoFisher Scientific	Cat#A35508
POU5F1 crRNA	ThermoFisher Scientific	Cat#CRISPR777205_CR
SOX2 crRNA	ThermoFisher Scientific	Cat#CRISPR1081382_CR
NANOG crRNA	ThermoFisher Scientific	Cat#CRISPR850052_CR
TFAP2C crRNA	ThermoFisher Scientific	Cat#CRISPR906394_CR

**Recombinant DNA**		

pGG195/GATA3mKO2	This study	N/A
CML32	This study	N/A
px459_SpCas9-2A-Puro	Addgene	Cat#62988
PBase	Guo et al., 2009	N/A
AAVS1-Cas9 targeting vector	Gift from Kosuke Yusa	N/A
pGG150-hNanog	This study	N/A
PB-CAG-Tet3G-IZ	This study	N/A

**Software and algorithms**		

STAR	Dobin et al., 2013	https://github.com/alexdobin/STAR
htseq-count	Anders et al., 2014	https://htseq.readthedocs.io/en/master/
DESeq	Anders and Huber, 2010	https://www.huber.embl.de/users/anders/DESeq/
FactoMineR	Lê et al., 2008	http://factominer.free.fr/
TF annotations		http://bioinfo.life.hust.edu.cn/AnimalTFDB/
Samtools	Li et al., 2009	http://samtools.sourceforge.net/
MFuzz	Kumar and Futschik, 2007	https://bioconductor.org/packages/release/bioc/html/Mfuzz.html
R	N/A	https://www.R-project.org/
MASS	Venables and Ripley, 2002	https://cran.r-project.org/web/packages/MASS/index.html
Genome and Genome annotation	Ensembl 96	http://apr2019.archive.ensembl.org/index.html
gplots	N/A	https://cran.r-project.org/web/packages/gplots/index.html
plot3D	N/A	https://cran.r-project.org/web/packages/plot3D/index.html
Cell Ranger v3.1.0	Zheng et al. (2017)	https://support.10xgenomics.com/single-cell-gene-expression/software/downloads/latest
Seurat v3.1.5	Stuart et al. (2019)	https://satijalab.org/seurat/
R v4.0.0	N/A	https://www.R-project.org/
ggplot2		https://ggplot2.tidyverse.org/

### Resource availability

#### Lead contact

Further information and requests for resources and reagents should be directed to and will be fulfilled by the lead contact, Ge Guo, g.guo@exeter.ac.uk

#### Materials availability

All stable reagents generated in this study are available from the lead contact without restriction except for human embryo derived cell lines for which permission must be requested from the UK Stem Cell Steering Committee and a Materials Transfer Agreement completed.

#### Data and code availability

The RNaseq datasets reported in this paper are deposited in Gene Expression Omnibus with accession codes: RNaseq, GEO: GSE166401 and GEO: GSE167089; scRNaseq, GEO: GSE166422.

### Experimental model and subject details

#### Human embryos

Supernumerary frozen human embryos were donated with informed consent by couples undergoing *in vitro* fertility treatment. Use of human embryos in this research is approved by the Multi-Centre Research Ethics Committee, approval O4/MRE03/44, and licensed by the Human Embryology & Fertilization Authority of the United Kingdom, research license R0178.

#### Cell cultures

Cell lines are listed in the Key Resources Table. Cell lines were cultured in humidified incubators at 37°C in 7% CO_2_ and 5% O_2_. Cell were cultured without antibiotics and tested negative for mycoplasma by periodic PCR screening.

### Method details

#### Human embryos

Supernumerary frozen blastocysts (mixture of E5 and E6) were thawed and cultured in N2B27 medium under mineral oil. The majority of embryos were cultured for 24 hours for development to fully expanded late blastocysts (E6 or E7) assessed by zona thinning, estimated number of cells in the mural TE, and size of ICM. On rare occasions when embryos were already fully expanded on thawing, they were processed immediately. Embryos that failed to expand fully were not used.

Immunosurgery was performed as described (Guo et al., 2016). In occasional cases when lysis and removal of the trophectoderm could not be assured, embryos were excluded from the study.

For microdissection, embryos were first labeled by incubation with WGA conjugate for 10 minutes and washed in pre-equilibrated N2B27. Mural trophectoderm was excised using a finely drawn Pasteur pipette of internal diameter just larger than the embryo. ICM and polar trophectoderm were dissociated using accutase for 10 minutes, followed by aspiration of individual cells or small clusters into a drop of N2B27 using a finely drawn Pasteur pipette of diameter just larger than a cell.

#### Culture of ICMs and embryo cells

Isolated ICMs were placed intact on laminin-coated plates in N2B27 medium with or without inhibitors. For time-lapse imaging and confocal microscopy, immunosurgically isolated ICMs, microdissected ICMs with polar trophectoderm, or dissociated ICM and polar trophectoderm were cultured on Ibidi 24-well μ-plates coated with recombinant Laminin-111 E8 (Taniguchi et al., 2009). Rho associated kinase inhibitor Y-27632 was added to dissociated cell cultures.

After 16-20h anti-SUSD2 was added to the medium (1:50) for live naive epiblast staining (Bredenkamp et al., 2019a) and the positions of cells were registered. Cells were imaged every day until cultures were fixed for immunostaining after 96h. Images were processed in FIJI; images from the same time-point were set to the same brightness and contrast followed by a rolling-ball background correction.

#### hPSC culture

##### Naive stem cells

Chemically reset (cR), embryo-derived (HNES1) and reprogrammed (niPSC) naive stem cells were propagated in N2B27 with PXGL [1μM PD0325901 (P), 2μM XAV939 (X), 2μM Gö6983 (G) and 10ng/mL human LIF (L)] on irradiated MEF feeders as described (Bredenkamp et al., 2019b). Y-27632 and Geltrex (0.5μL per cm^2^ surface area; hESC-Qualified, Thermo Fisher Scientific, A1413302,) were added during replating. Cultures were passaged by dissociation with Accutase (Biolegend, 423201) every 3-5 days.

##### Conventional hPSCs

Conventional primed hPSCs (H9, Shef6) were propagated on Geltrex in Essential 8 (E8) medium made in-house (Chen et al., 2011) or in AFX medium (N2B27 basal medium with 5ng/mL Activin A, 5ng/mL FGF2 and 2μM XAV).

#### Differentiation

Human naive cells were plated in PXGL with Y-27632 on Geltrex or Laminin at a 1:4 to 1:6 ratio. The next day, cultures were washed twice with PBS and medium exchanged to N2B27 with chemical inhibitors or cytokines. Medium was refreshed every day until assaying. Human primed cells were plated in AFX medium on Geltrex at 1:6 to 1:10 ratio and exchanged to assay conditions similarly to naive cells. Medium was refreshed every day until assaying. Concentrations used in this assay: PD03 1μM, A83-01 1μM, BMP2 50ng/mL, LDN-193189 100nM, Activin A 20ng/mL. For formation of cysts, naive cells were dispensed in round-bottom non-adherent 96-well plates and cultured in suspension in PD+A83 for 3-5 days.

#### Capacitation of human naive cells

Cells were passaged once without feeders in PXGL medium then exchanged into N2B27 containing 2μM XAV for 10 days (Rostovskaya et al., 2019), followed by propagation in AFX medium.

#### Generation of GATA3:mKO2 reporter cell line

The pGG195/GATA3:mKO2 targeting vector was designed to insert an iresmKO2-FRT-PGKNeobPA cassette following the stop codon of *GATA3*. 1x10^6^ HNES1 naive cells were transfected with 3μg pGG195/GATA3mKO2 and 3ug px459/GATA3 gRNA using 100 μl Neon transfection kit. G418 (250μg/mL) selection was applied 2 days after transfection for 4 days. Cells were then harvested and transfected with CAGGS-Flp plasmid and plated in 2x10cm plates. Clones were picked 7 days after transfection and assayed for mKO2 expression in PXGL and PD03. Genomic DNA was prepared and correctly targeted heterozygous clones were confirmed by PCR amplification of the targeted junction and sequencing.

#### Trophoblast stem cell culture

After 3-5 days treatment with PD only or PD+A83, cultures were passaged onto MEF or collagen IV-coated dishes in trophoblast stem cell culture medium (Okae et al., 2018); DMEM/F12 supplemented with 0.1mM 2-mercaptoethanol, 0.2% FBS, 0.3% BSA, 1% ITS-X supplement, 1.5mg/ml L-ascorbic acid, 50 ng/ml EGF, 2 μM CHIR99021, 1.0 μM A83-01, 0.8mM VPA and 5 μM Y-27632. Cells were passaged by dissociation with TrypLE. Differentiation was induced as described (Okae et al., 2018).

#### Inducible NANOG expression

HNES1/GATA3:mKO2 reporter cells were co-transfected with two *Piggybac* vectors carrying a *Tet3G*-inducible *NANOG* expression cassette and a *CAG-Tet3G-IresZeocin* cassette together with *PBase* plasmid. Two days after transfection, zeocin (50 μg/mL) was applied for 5 days and individual clones were picked after 7 days. *NANOG* expression was induced with 10-20 ng/mL doxycycline and assayed by qRT-PCR.

#### CRISPR/Cas9 knockout

##### Knockout by Cas9/gRNA RNP transfection

TrueGuide synthetic crRNAs were purchased from Thermo Fisher Scientific, reconstituted and annealed with tracrRNA in RNA annealing buffer to generate double-stranded RNA duplex. The annealed RNA duplex was diluted in RNA storage buffer to 10 μM stock. For each transfection, 1.2 μl of the 10μM gRNA duplex was mixed with 300 ng Cas9 protein and incubated at room temperature for 15 min before transfection. 10μL Neon transfection kit was used to transfect 1-1.5x10^5^ cells at 1150V, 30ms, 2 pulses. After transfection cells were plated without feeders in PXGL medium with ROCK inhibitor (Y-27632, 10 μM). After 24 hours medium was exchanged to N2B27 or other differentiation assay medium for 4 days.

##### Knockout by gRNA plasmid transfection in Cas9 expressing naive cells

gRNA oligos (Table S1) were synthesized and annealed to double-stranded DNA and cloned behind a U6 promoter (CML32) into a *Piggybac (PB*) vector containing a puromycin resistance gene. gRNA-expression plasmids were transfected together with *PBase* plasmid into HNES1 GATA3:mKO2 cells that had been engineered to constitutively express *Cas9* from the *AAVS1* genomic locus. Following transfection, cells were plated without feeders in PXGL with Y-27632 for 2 days then exchanged to medium for differentiation assay. Puromycin (0.5 μg/mL) was applied for at least 3 days to select cells with PB plasmid integration.

#### Reverse transcription and real-time PCR

Total RNA was extracted using ReliaPrep kit (Promega, Z6012) and cDNA synthesized with GoScript reverse transcriptase (Promega, A5004) and oligo(dT) adaptor primers. TaqMan assays (Thermo Fisher Scientific) and Universal Probe Library (UPL) probes (Roche Molecular Systems) were used to perform gene quantification.

#### Immunostaining

Cells were fixed with 4% PFA for 10 min at room temperature and blocked/permeabilised in PBS with 0.1% Triton X-100, 5% Donkey serum for 30 min. Incubation with primary antibodies was overnight at 4°C. Wash was in 0.1% Triton X-100 twice, 10 min each time. Secondary antibodies were added for 1 h at room temperature. Whole embryo and embryo explant staining was performed as described (Guo et al., 2016)

#### Microscopy

Wide field images were taken using Leica DMI3000. Confocal images were taken using a Leica SP-2 system. Time-lapse images were taken using a Leica DMI6000 Matrix system fitted with a controlled temperature and CO_2_ chamber. Images were analyzed with ImageJ software.

#### Flow cytometry

Flow cytometry was carried out on CyAn ADP (Beckman Coulter) or BD LSR Fortessa instruments (BD Biosciences) with analysis using FlowJo software. DAPI staining was used to exclude the dead cell population.

#### Transcriptome sequencing

For bulk RNA seq, total RNA was extracted from two biological replicate cultures of each cell line and time point using TRIzol/chloroform (Thermo Fisher Scientific, 15596018), and RNA integrity assessed by Qubit measurement and RNA nanochip Bioanalyzer. Ribosomal RNA was depleted from 1 μg of total RNA using Ribozero (Illumina kit). Sequencing libraries were prepared using the TruSeq RNA Sample Prep Kit (RS-122-2001, Illumina). Sequencing was performed on the Novaseq S1 or S2 platform (Illumina).

For 10x Genomics single cell RNaseq, cultures were dissociated with TrypLE Express Enzyme at 37°C for 10 min. Single cell populations were sorted using a flow cytometer based on forward/side scatter into PBS with 0.04% BSA. Single cell libraries were created using Chromium Single Cell 3′ Reagent Kits and sequenced on a Novaseq 6000 sequencer. Approximately 3000-5000 cells were captured for each time point.

### Quantification and statistical analysis

Alignment was performed using the Genome build GRCh38 and STAR (Dobin et al., 2013) were used for aligning reads. Ensembl release 96 was used to guide gene annotation. After removal of inadequate samples, we quantified alignments to gene loci with htseq-count (Anders et al., 2014) based on annotation from Ensembl 96. Principal component, differential expression and cluster analyses were performed based on log2 expression values computed with custom scripts, in addition to the Bioconductor packages DESeq (Anders and Huber, 2010), FactoMineR (Lê et al., 2008) and MFuzz (Kumar and Futschik, 2007). Gene density that contributed to PCA plots were calculated using kernel density estimation (MASS R package; Venables and Ripley, 2002)

For global analyses, we considered only genes with log2 expression > 0 (unless otherwise indicated) in at least one condition, not expressed genes were always omitted. Euclidean distance and average agglomeration methods were used for cluster analyses unless otherwise indicated. Human transcription factor and co-factors were downloaded from http://bioinfo.life.hust.edu.cn/AnimalTFDB/. Time courses for H1.ESC, H1.EPSC and hiEPSC (Gao et al., 2019) were downloaded from array express and re-aligned.

For 10x analyses Cellranger-3.1.0 count (Zheng et al., 2017) was run using default parameters and Cellranger’s prebuilt human reference genome ‘refdata-cellranger-GRCh38-3.0.0’, producing counts matrices which were merged and analyzed using Seurat 3.1.5 (Stuart et al., 2019) in R. A bimodal distribution of the number of genes expressed per cell was observed therefore all cells expressing fewer than 3000 genes or more than 10% mitochondrial genes were removed. The remaining cells were log-normalized via the division of each cell’s feature counts by the cell’s total counts, which were then multiplied by a scale factor of 10,000 and finally natural-log (log1p) transformed. The top 2000 most variably expressed genes within the dataset were identified, scaled, and centered for use in initial dimensionality reduction with PCA prior to further non-linear dimensionality reduction using UMAP. Cells visualized in UMAP plots were colored according to individual marker gene expression values and similarity to cell type-specific gene expression signatures, scored using Seurat’s AddModuleScore function. Z-scores were used to plot heatmaps and dotplots of marker expression.

The Cynomolgus monkey dataset (Nakamura et al., 2016) was kindly provided by Dr. Nakamura as an RPM table.

Expression values of ESC cultured in different human naive/primed conditions and pre-implantation embryo single cell RNaseq were downloaded from (Stirparo et al., 2018). Embryo cells expressing less than 6000 genes with log2 expression > 1.5 were excluded. EPI, HYP and TE markers for cell stratification were selected according to Stirparo et al. (2018).

Human *in vitro* embryo single cells were downloaded from Xiang et al., 2019. High variable genes across EPI cells were computed according to the methods described (Boroviak et al., 2018; Stirparo et al., 2018). A non-linear regression curve was fitted between average log2 FPKM and the square of coefficient of variation (log CV^2^); then, specific thresholds were applied along the x axis (average log_2_ expression) and y axis (log CV^2^) to identify the most variable genes. Cells expressing less than 6000 genes with log2 expression > 1.5 were excluded from analysis.

Human amnion-like genes were downloaded from Zheng et al. (2019) (their Table S1, AMLC genes) and we selected only genes > 1 expression in amnion. Early and late amnion genes in cynomolgus monkey were downloaded from Ma et al. (2019) (their Table S6).

## Supplemental information

Document S1. Figures S1–S7 and Table S1

Document S2. Article plus supplemental information
