# Supplementary material for: Human naive epiblast cells possess unrestricted lineage potential
Source: Cell Stem Cell. 2021 Jun 3;28(6):1040–1056.e6. doi: 10.1016/j.stem.2021.02.025 (PMC8189439; doi:10.1016/j.stem.2021.02.025)
Supplement: Document S1. Figures S1–S7 and Table S1 [file mmc1.pdf]

Cell Stem Cell, Volume *28*

Supplemental Information

Human naive epiblast cells possess unrestricted lineage potential

Ge Guo, Giuliano Giuseppe Stirparo, Stanley E. Strawbridge, Daniel Spindlow, Jian Yang, James Clarke, Anish Dattani, Ayaka Yanagida, Meng Amy Li, Sam Myers, Buse Nurten Özel, Jennifer Nichols, and Austin Smith

SUPPLEMENTAL TABLE

Table S1 gRNA sequences for targeting in Cas9 expressing naïve cells, Related to Star Methods

gRNA target	***Sequence***
POU5F1-g1	GCCACCAAATAGAACCCCCA
POU5F1-g2	GCCACCAAATAGAACCCCCA
SOX2-g1	GTTATAAATACCGGCCCCGG
SOX2-g2	GCAGGGCGCTCACGTCGTAG
NANOG-g1	GGTTCACCAGGCATCCCTGG
NANOG-g2	GGTCGCAAAAAAGGAAGACA
TFAP2C-g1	ATATTCGGCGACTCCAGTGT
TFAP2C-g2	GCTTAAATGCCTCGTTAC
YAP-g1	GACGTTCATCTGGGACAGCA
TAZ-g1	TACGAGCTCATCGAGAAGCG
GFP-g1	GAGCTGGACGGCGACGTAAA

SUPPLEMENTAL FIGURE LEGENDS

Figure S1. Trophectoderm formation, Related to Figure 1

A. qRT-PCR assay for hypoblast markers after 5 days in indicated conditions.
B. qRT-PCR assay for naïve and trophectoderm markers after 5 days in indicated conditions.
C. qRT-PCR assay for trophectoderm markers after 5 days in N2B27 or PD03 of human naïve PSC lines; HNES1, cR-NCRM2 and cR-Shef6
D. qRT-PCR assay for core pluripotency and trophectoderm marker expression in mouse ES cells cultured for 3 days in indicated conditions.
E. Phospho-Smad2/3 immunoblot for conventional H9 hPSCs and naïve cells in indicated conditions.
F. Phospho-Smad2/3 immunoblot for naïve cells in absence or presence of A83
G. Phase and fluorescence images of GATA3:mKO2 cells in PXGL with or without A83 for 4 passages. Scale bar, 100µM
H. Flow cytometry analysis of GATA3:mKO2 cells in PXGL or in PXGL plus A83 for 4 passages.
I. qRT-PCR assay for *GATA2* and *GATA3* expression in human naïve cells cultured in PXGL with A83. X-axis indicates passages in PXGL with A83.
J. Flow cytometry analysis of GATA3:mKO2 cells in N2B27 or with A83 for 3 days.
K. Phase contrast and fluorescence time lapse stills of GATA3:mKO2 cells in PD+A83. (See also Supplemental Movies 1 and 2).

Figure S2. Trophectoderm differentiation and cytotrophoblast stem cells, Related to Figure 2

A. Wide field images of cysts formed in adherent culture in PD+A83 immunostained for aPKCι and PAR6B.
B. Two examples of suspension cysts outgrown for 3 days in N2B27 and immunostained for CK7 and extravillous trophoblast marker HLA-G.
C. qRT-PCR analysis of gene expression in placenta and naïve stem cell derived TSCs. Naive stem cell derived TSCs include two independent cultures derived from HNES1 and one each from naïve iPSC lines niPSC2 and niPSC4. CT27 is a placental cytrophoblast TSC line (Okae et al, 2018). Error bars from technical duplicates.
D. qRT-PCR analysis of gene expression during differentiation of placenta and naïve stem cell derived TSCs using syncitiotrophoblast (SCT) and extravillous trophoblast (EVT) protocols. Error bars from technical duplicates.

Figure S3. Whole transcriptome analysis, Related to Figure 3

A. One-way hierarchal clustering of early blastocyst (E5) single cells (Petropoulos et al., 2016) computed with lineage marker genes (Stirparo et al., 2018).
B. One-way hierarchal clustering of E6 and E7 single cells (Petropoulos et al., 2016) computed with lineage marker genes (Stirparo et al., 2018).
C. PCA computed for all the genes expressed in early ICM (cluster 4, FIG.S2A), early TE (cluster 5, Figure S2A), epiblast (Stirparo et al., 2018) and late TE (cluster 2, Figure S2 B, C, D). In red, cells expressing more than 6000 genes.
D. PCA computed as in C for filtered cells expressing >6000 genes (log2expression >0, n=18694)

E. PCA for filtered cells, computed with differentially expressed genes in human embryo (n= 4507).
F. As Figure 3B for cR-H9 cells.
G. PCA computed with all orthologues (average macaque dataset, ICM, EPI, postE, postL, early TE, late TE and post PA.TE, Nakamura et al., 2016) of expressed protein coding genes (log2 expression in time-course > 0 & orthologues, n= 12992).

Figure S4. Single cell analysis, Related to figure 4

A. Expression of early trophectoderm genes coloured on Figure 4A UMAP
B. Expression of cytotrophoblast, syncitiotrophoblast and extravillous trophoblast lineage markers on Figure 4A UMAP
C. Expression of mid (E9-10) and late (E12-14) post-implantation epiblast markers on Figure 4A UMAP
D. Expression of additional hypoblast markers on Figure 4E UMAP
E. Immunostaining of cells cultured in PD+A83 for 24h followed by 48h in A83 or N2B27.

Figure S5. Genetic perturbations, Related to Figure 5

A. Alkaline phosphatase (AP) staining after indicated Cas9/gRNA RNP transfection and culture in PXGL on MEF for 4 days. Separate experiments were performed on parental HNES1 or HNES1-GATA3:mKO2 cells. Controls were transfected with GFP gRNA.
B. AP staining after indicated gRNA plasmid transfection in Cas9 expressing HNES1-GATA3:mKO2 cells. Cells were maintained in PXGL on MEF with puromycin selection for 7 days. Controls were transfected with GFP gDNA.
C. Flow cytometry analysis of GATA3:mKO2 cells after gRNA plasmid transfection and culture for 4 days in N2B27 alone or with A83.
D. Flow cytometry analysis of GATA3:mKO2 expression after Cas9 RNP transfection with GFP or TFAP2C gRNA.

Figure S6. Fates of naïve versus primed stem cells, Related to Figure 6

A. Flow cytometry analysis of naïve GATA3:mKO2 cells in indicated culture conditions for three days. LDN, BMP receptor inhibitor LDN-193189.
B. Phospho-Smad1/5 immunoblot on naïve cells and primed HNES1 cells in indicated conditions. P, PD03; B, BMP2, number indicates BMP2 concentration, ng/ml; L, human LIF.
C. Flow cytometry analysis of naïve and primed GATA3:mKO2 cells in indicated culture conditions for 5 days.
D. qRT-PCR assay of trophectoderm and amnion markers in human naïve and primed cell differentiated in PD+A83 with or without LDN for 5 days. Error bars from technical duplicates.
E. qRT-PCR assay for neural markers in naïve or primed PSCs differentiated in indicated conditions for 5 days with or without LDN. Error bars from technical duplicates.
F. PCA of human naïve and conventional primed cells (Stirparo et al., 2018), naïve cells from this study, and hEPSCs (Gao et al., 2019; Yang et al., 2017) computed using most variable genes, log2expression > 1, cv>0.5, n=3510.
G. 3D PCA of human naïve and conventional primed cells, naïve cells from this study, hEPSCs, and human embryo in vitro development (Xiang et al., 2019), computed with variable genes in embryo development, n=1517.

H. Two-dimensional kernel density estimation of amnion genes from human differentiation in vitro (expression >1, Zheng et al., 2019) in Figure 6D PCA.
I. Two-dimensional kernel density estimation of amnion genes from *macaca* embryo cultures (Ma et al., 2019) in Figure 6D PCA.
J. Density of placental cytotrophoblast TSC (Okae, 2019)(left) and AME (right) enriched genes in Figure 6F PCA.
K. Violin plot of z-score for TSC- and AME-enriched genes in differentiated hEPSCs (group 1) and naïve stem cell derived and placental TSCs (group 2) (see Figure 6F).
L. Bootstrap Spearman correlation (iteration 100, number of genes=50) of naïve or EPSC differentiation timecourses with differentiated populations induced by BAP treatment.
M. As Figure 6H with addition of BAP cell expression values.

Figure S7. Plasticity of human embryo pre-implantation epiblast, Related to Figure 7

A. Phase images of human blastocysts cultured for 24h and classified as fully expanded (E6 or E7) for use in this study.
B. Phase contrast and immunofluorescence images of immunosurgically isolated ICM explants after 5 days in N2B27 or PD+A83.
C. Time lapse stills at 0h and 48h with endpoint immunostaining of immunosurgically isolated ICM explants in PD+A83 plus LDN.
D. Images of microdissected E6 embryo with epiblast labelled by live cell immunostaining for SUSD2-FITC after culture for 16h. Arrow points to SUSD2 negative cells. Subsequent images show that cells persist as SUSD2 staining fades.
E. Immunostaining of SUSD2 labelled microdissected ICM with polar trophectoderm in Figure 7C after 5 days in PD+A83.
F. WGA (Alexa 594 conjugate) labelling of intact fully expanded blastocysts. Scale bar, 200μM.

Figure S1

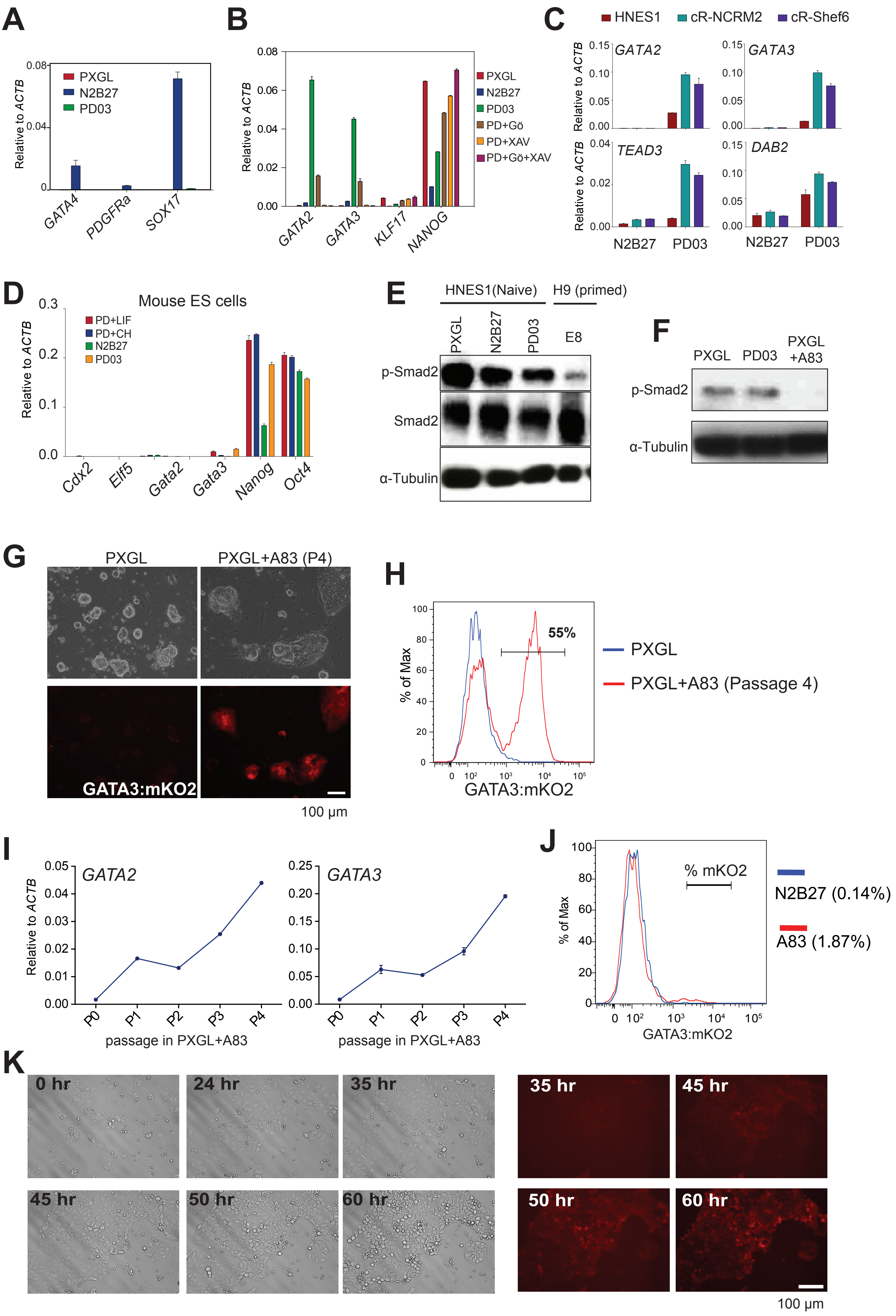

A
Relative to ACTB
PXGL
N2B27
PD03
GATA4
PDGFRa
SOX17
B
Relative to ACTB
PXGL
N2B27
PD03
PD+Gö
PD+XAV
PD+Gö+XAV
GATA2
GATA3
KLF17
NANOG
C
HNES1
cR-NCRM2
cR-Shef6
GATA2
GATA3
TEAD3
DAB2
N2B27
PD03
D
Mouse ES cells
PD+LIF
PD+CH
N2B27
PD03
Cdx2
Elf5
Gata2
Gata3
Nanog
Oct4
E
HNES1(Naive)
H9 (primed)
PXGL
N2B27
PD03
E8
p-Smad2
Smad2
α-Tubulin
F
PXGL
PD03
PXGL +A83
p-Smad2
α-Tubulin
G
PXGL
PXGL+A83 (P4)
GATA3:mKO2
100 µm
H
55%
% of Max
GATA3:mKO2
PXGL
PXGL+A83 (Passage 4)
I
GATA2
GATA3
passage in PXGL+A83
J
% mKO2
% of Max
GATA3:mKO2
N2B27 (0.14%)
A83 (1.87%)
K
0 hr
24 hr
35 hr
45 hr
50 hr
60 hr
100 µm

Figure S2

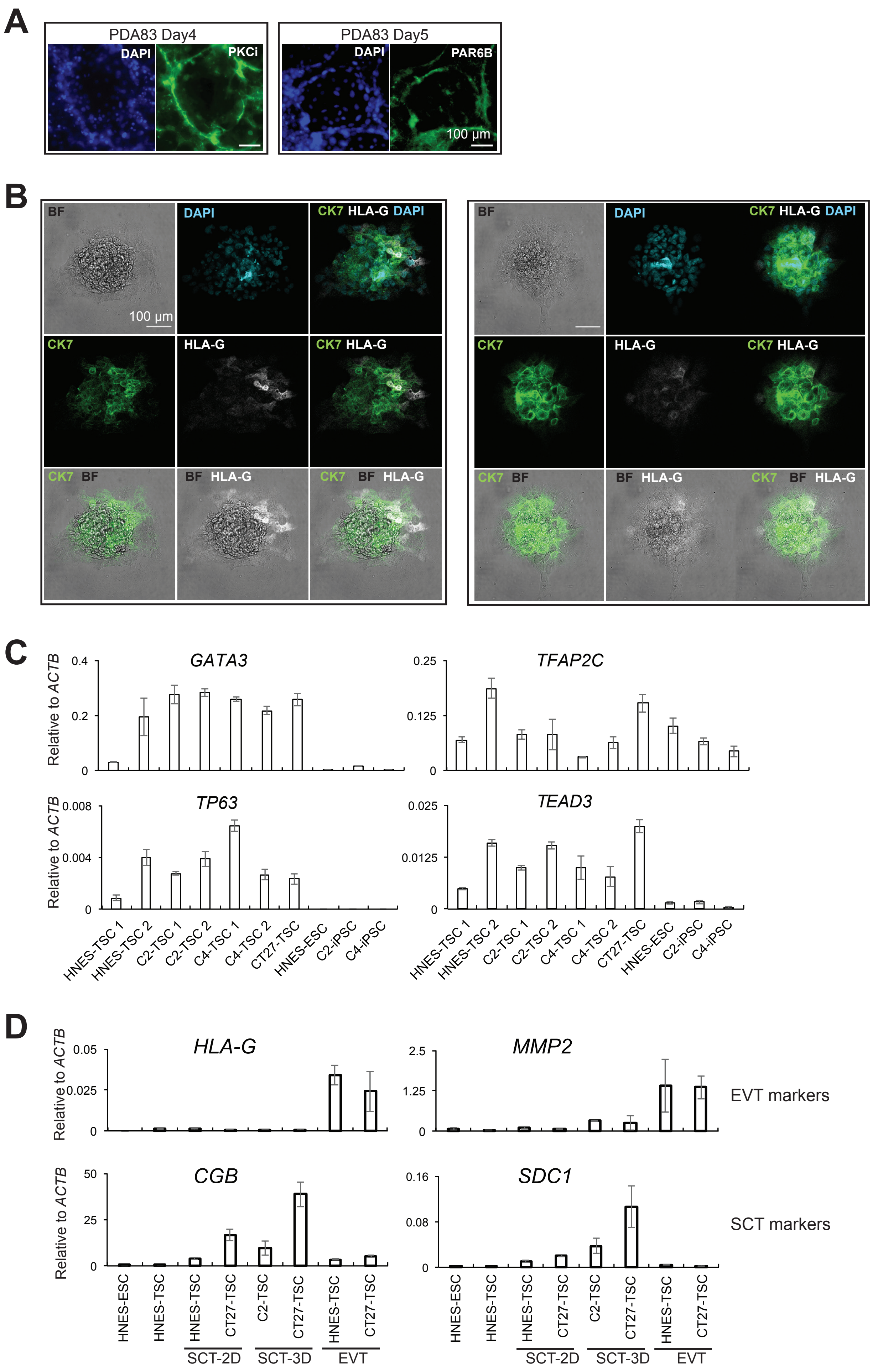
A
PDA83 Day4
DAPI
PKCi
PDA83 Day5
DAPI
PAR6B
100 µm
B
BF
DAPI
CK7 HLA-G DAPI
100 µm
CK7
HLA-G
CK7 HLA-G
CK7 BF
BF HLA-G
CK7 BF HLA-G
C
GATA3
TFAP2C
TP63
TEAD3
Relative to ACTB
HNES-TSC 1
HNES-TSC 2
C2-TSC 1
C2-TSC 2
C4-TSC 1
C4-TSC 2
CT27-TSC
HNES-ESC
C2-iPSC
C4-iPSC
D
HLA-G
MMP2
EVT markers
CGB
SDC1
SCT markers
HNES-ESC
HNES-TSC
HNES-TSC
CT27-TSC
C2-TSC
CT27-TSC
HNES-TSC
CT27-TSC
SCT-2D
SCT-3D
EVT

Figure S3

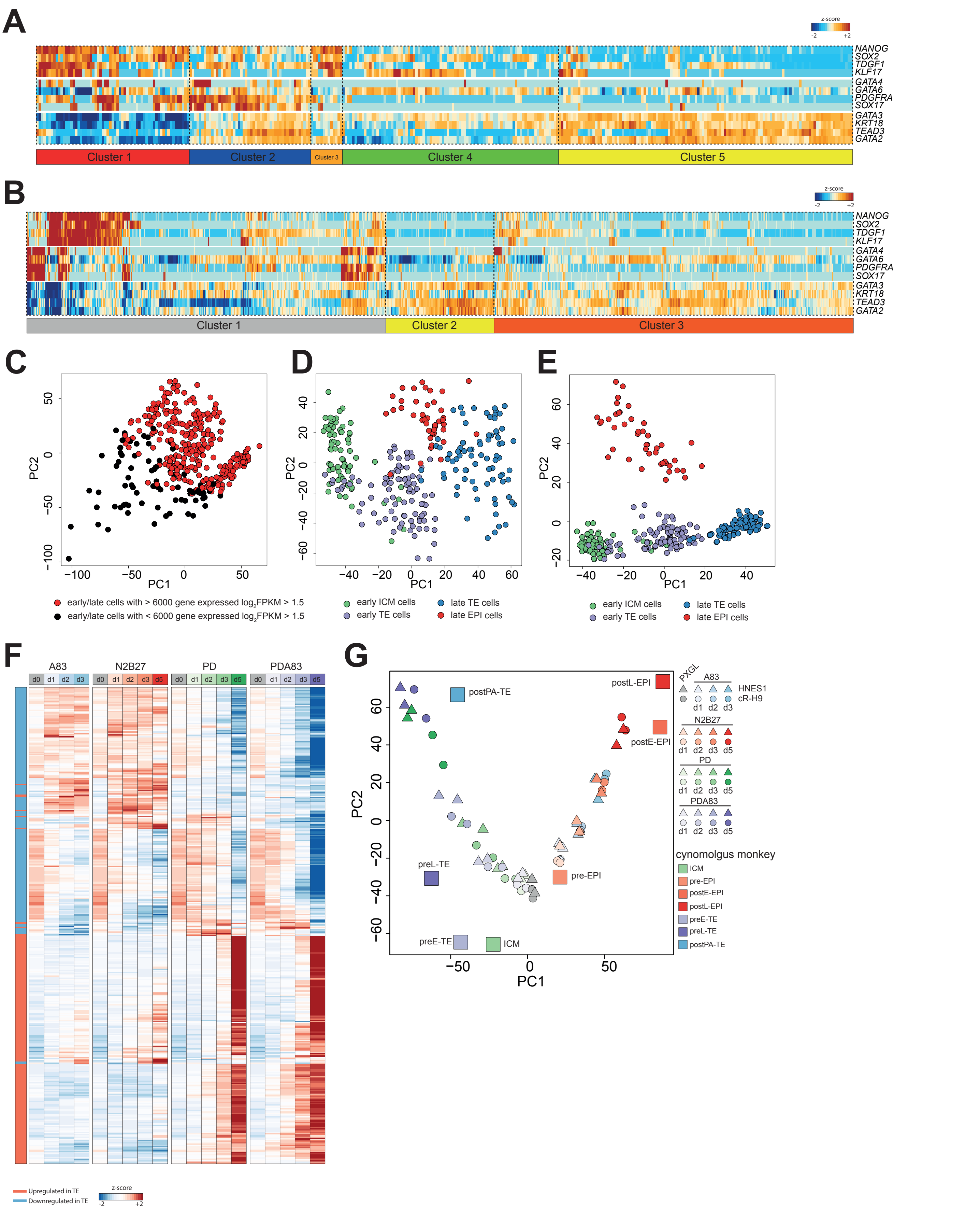
A
B
C
D
E
F
G
NANOG
SOX2
TDGF1
KLF17
GATA4
GATA6
PDGFRA
SOX17
GATA3
KRT18
TEAD3
GATA2
Cluster 1
Cluster 2
Cluster 3
Cluster 4
Cluster 5
z-score
early/late cells with > 6000 gene expressed log2FPKM > 1.5
early/late cells with < 6000 gene expressed log2FPKM > 1.5
early ICM cells
early TE cells
late TE cells
late EPI cells
PC1
PC2
A83
N2B27
PD
PDA83
d0
d1
d2
d3
d5
Upregulated in TE
Downregulated in TE
PXGL
HNES1
cR-H9
postPA-TE
postL-EPI
postE-EPI
pre-EPI
preL-TE
preE-TE
ICM
cynomolgus monkey

Figure S4

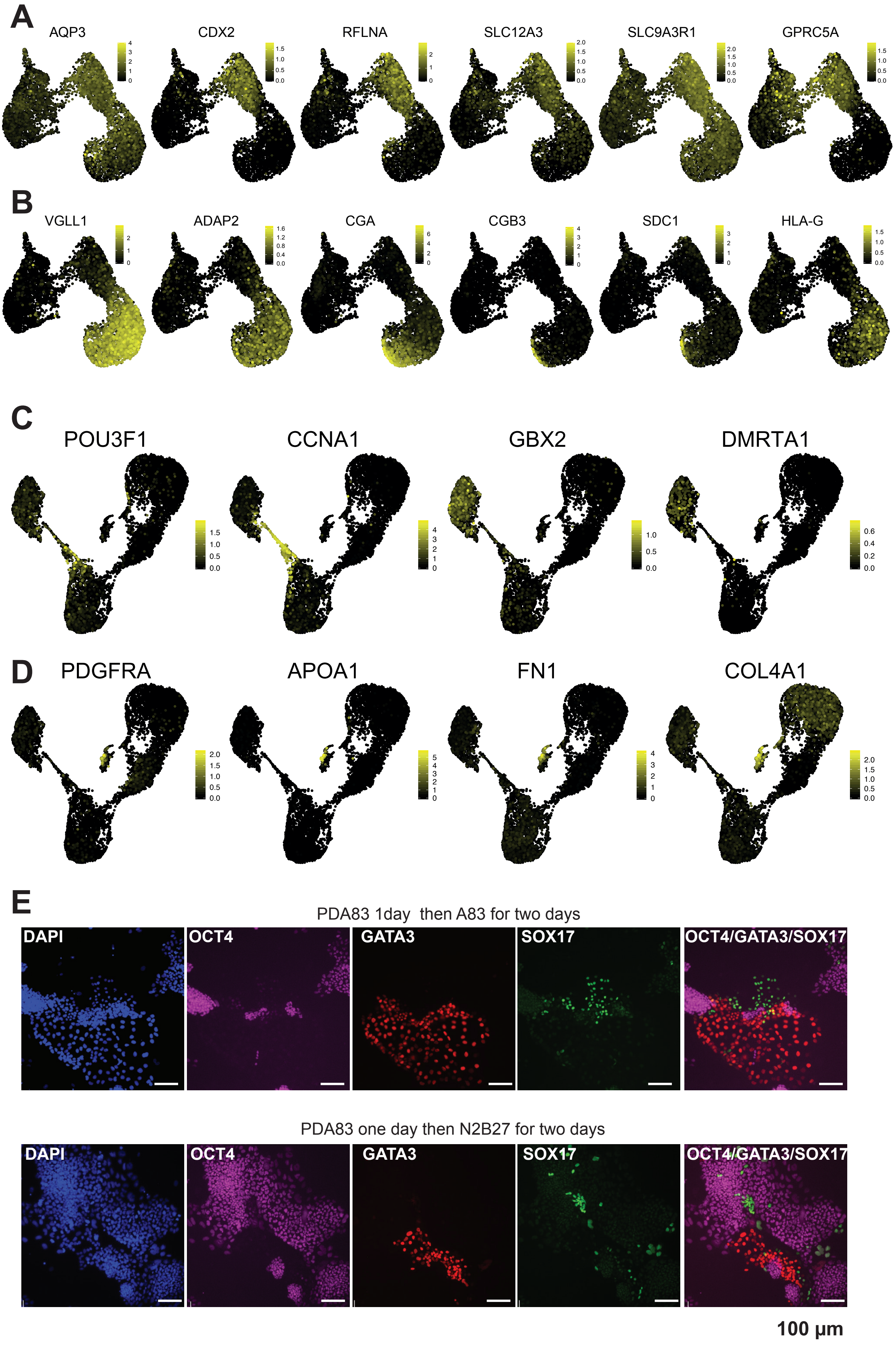
A
AQP3
CDX2
RFLNA
SLC12A3
SLC9A3R1
GPRC5A
B
VGLL1
ADAP2
CGA
CGB3
SDC1
HLA-G
C
POU3F1
CCNA1
GBX2
DMRTA1
D
PDGFRA
APOA1
FN1
COL4A1
E
PDA83 1day then A83 for two days
DAPI
OCT4
GATA3
SOX17
OCT4/GATA3/SOX17
PDA83 one day then N2B27 for two days
DAPI
OCT4
GATA3
SOX17
OCT4/GATA3/SOX17
100 µm

Figure S5

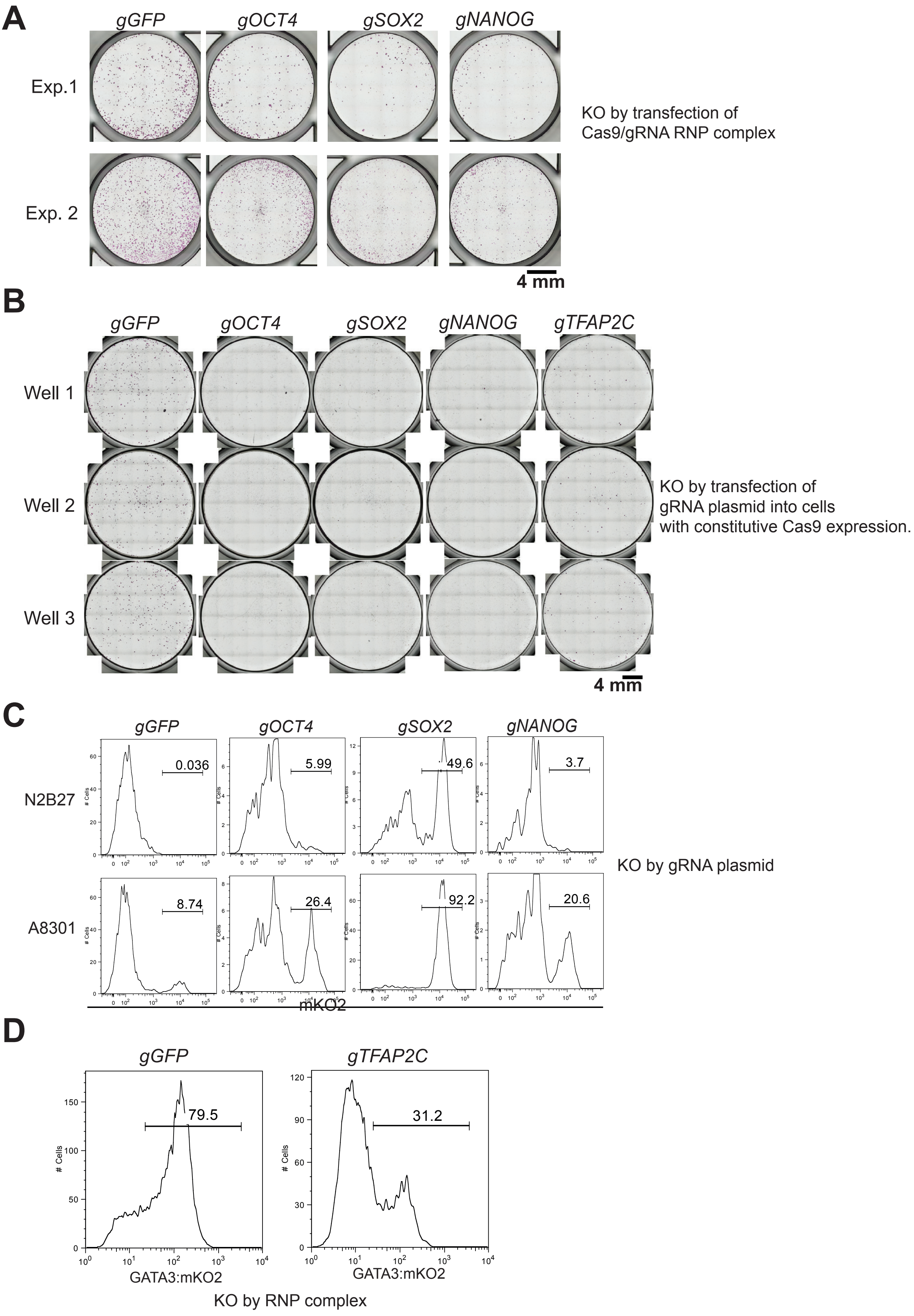
A
gGFP
gOCT4
gSOX2
gNANOG
Exp.1
Exp. 2
KO by transfection of
Cas9/gRNA RNP complex
4 mm
B
gGFP
gOCT4
gSOX2
gNANOG
gTFAP2C
Well 1
Well 2
Well 3
KO by transfection of
gRNA plasmid into cells
with constitutive Cas9 expression.
4 mm
C
gGFP
gOCT4
gSOX2
gNANOG
N2B27
A8301
0.036
5.99
49.6
3.7
8.74
26.4
92.2
20.6
Cells
mKO2
KO by gRNA plasmid
D
gGFP
gTFAP2C
79.5
31.2
Cells
GATA3:mKO2
KO by RNP complex

Figure S6

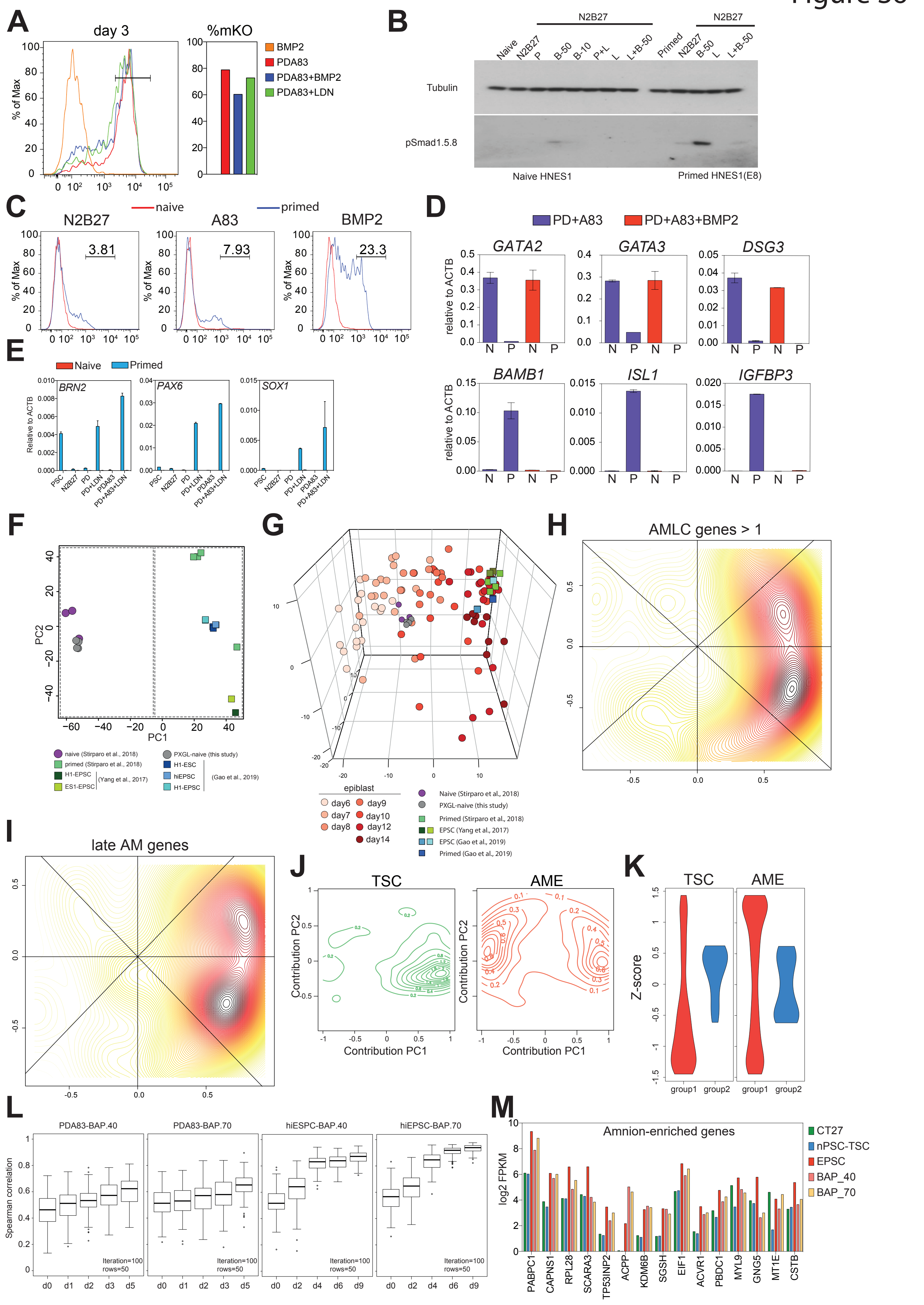

A
day 3
%mKO
% of Max
BMP2
PDA83
PDA83+BMP2
PDA83+LDN
B
N2B27
Naive
N2B27
P
B-50
B-10
P+L
L
L+B-50
Primed
N2B27
B-50
L
L+B-50
Tubulin
pSmad1.5.8
Naive HNES1
Primed HNES1(E8)
C
naive
primed
N2B27
A83
BMP2
3.81
7.93
23.3
D
PD+A83
PD+A83+BMP2
GATA2
GATA3
DSG3
BAMB1
ISL1
IGFBP3
relative to ACTB
E
Naive
Primed
BRN2
PAX6
SOX1
Relative to ACTB
PSC
N2B27
PD
PD+LDN
PDA83
PD+A83+LDN
F
PC1
PC2
naive (Stirparo et al., 2018)
primed (Stirparo et al., 2018)
H1-EPSC
ES1-EPSC
(Yang et al., 2017)
PXGL-naive (this study)
H1-ESC
hiEPSC
H1-EPSC
(Gao et al., 2019)
G
epiblast
day6
day7
day8
day9
day10
day12
day14
Naive (Stirparo et al., 2018)
PXGL-naive (this study)
Primed (Stirparo et al., 2018)
EPSC (Yang et al., 2017)
EPSC (Gao et al., 2019)
Primed (Gao et al., 2019)
H
AMLC genes > 1
I
late AM genes
J
TSC
AME
Contribution PC2
Contribution PC1
K
TSC
AME
Z-score
group1
group2
L
PDA83-BAP.40
PDA83-BAP.70
hiESPC-BAP.40
hiEPSC-BAP.70
Spearman correlation
Iteration=100
rows=50
M
Amnion-enriched genes
log2 FPKM
CT27
nPSC-TSC
EPSC
BAP_40
BAP_70
PABPC1
CAPNS1
RPL28
SCARA3
TP53INP2
ACPP
KDM6B
SGSH
EIF1
ACVR1
PBDC1
MYL9
GNG5
MT1E
CSTB

Figure S7

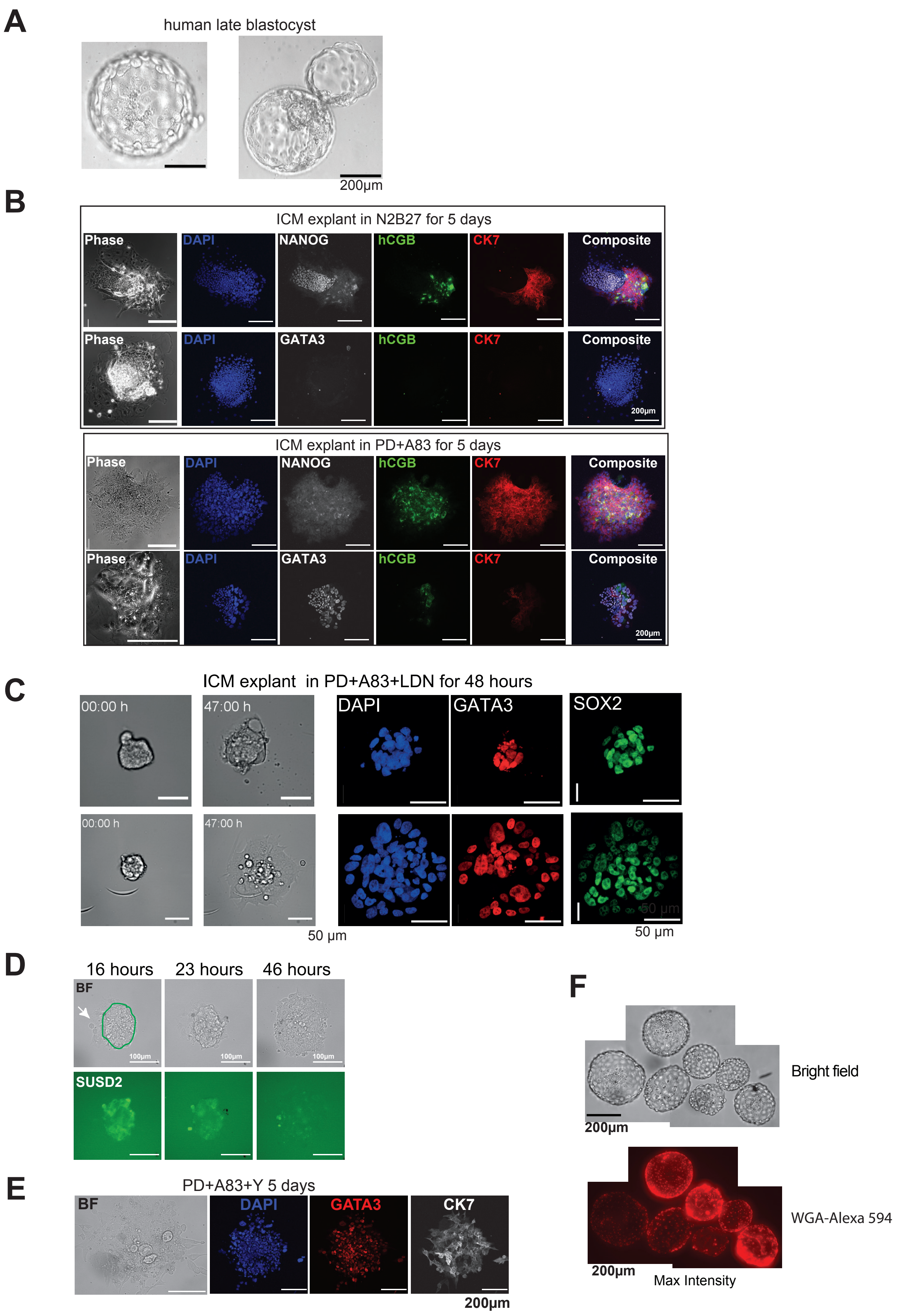

A
human late blastocyst
200µm
B
ICM explant in N2B27 for 5 days
Phase
DAPI
NANOG
hCGB
CK7
Composite
GATA3
200µm
ICM explant in PD+A83 for 5 days
C
ICM explant in PD+A83+LDN for 48 hours
00:00 h
47:00 h
SOX2
50 µm
D
16 hours
23 hours
46 hours
BF
SUSD2
100µm
E
PD+A83+Y 5 days
F
Bright field
WGA-Alexa 594
Max Intensity
